# Emergence of Nonuniform Strain-Induced Exciton Species in Bilayer Transition Metal Dichalcogenides

**DOI:** 10.1021/acsnano.5c09721

**Published:** 2026-06-30

**Authors:** Mohammadreza Daqiqshirazi, Thomas Brumme

**Affiliations:** † Theoretical Chemistry, Technische Universität Dresden, Bergstrasse 66c, 01069 Dresden, Germany; ‡ Center for Advanced Systems Understanding (CASUS), Untermarkt 20, D-02826 Görlitz, Germany; § Helmholtz Zentrum Dresden-Rossendorf, Bautzner Landstraße 400, D-01328 Dresden, Germany

**Keywords:** transition metal dichalcogenides, ab initio, bilayer, heterobilayer, wrinkle, nonuniform strain, localization

## Abstract

Full control over excitons in 2D materials is an important step toward their exploitation for applications. Strain modulation is one method that can be used to effectively control the movement of the excitons. Unfortunately, the effects of nonuniform strain in 2D materials are not yet well understood theoretically. However, these strain fields can be present in experiments in the form of wrinkles, bubbles, and folds, or even explicitly applied to 2D materials through prepatterned surfaces. The effects of these nonuniform strain fields on multilayers are even less studied because of the sheer size of these systems. In the present investigation, we study wrinkles that form in homo- and heterobilayers of 2D transition-metal dichalcogenides using density functional theory. We show that nonuniform strain could lead to the formation of spatially localized, momentum-direct, bright interlayer excitons IX^*KK*^ in homobilayers of transition-metal dichalcogenides such as WSe_2_ and WS_2_ and to exciton localization in transition-metal dichalcogenide heterobilayers. Our results also reveal that the spin angular momentum is changed due to the mixing of in- and out-of-plane states, which can explain the brightening of the formerly dark excitonic states under strain. Our results provide insights into a better understanding of the strain control of excitons in 2D materials.

## Graphical abstract

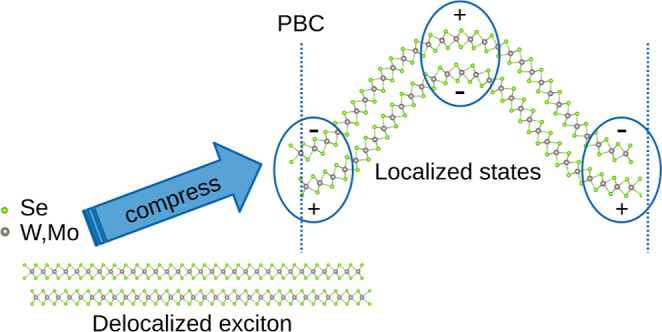

2D materials are an interesting class of materials that can differ significantly from their bulk crystals in their properties.^1,2^ One of the important features of these materials is their different or even opposing optoelectronic properties when compared to their bulk counterparts. These properties are indeed pivotal for the design of new devices. For example, Mo- and W-based transition metal dichalcogenides (TMDCs) undergo a transition from an indirect to a direct band gap when they are thinned down to their monolayers.^3,4^ This transition makes them suitable candidates for applications that require a direct band gap semiconductor, such as light-emitting diodes.^5,6^ In addition to the novel intrinsic properties of the 2D materials, these can also be modulated by structural manipulations. Structural variations can be achieved, for example, by interfacing different 2D materials to create heterostructures with unique properties,^7^ or by varying the twist angle between layers to form moiré (hetero)­structures.^8^ Furthermore, all these properties can be altered on demand utilizing external fields—especially strain provides a unique possibility to alter the electronic properties of 2D materials.^9,10^

Strain is widespread in 2D materials. It occurs not only during the synthesis or transfer of 2D materials onto substrates, but it can also be intentionally used to tune their electronic properties,^11–13^ a field known as straintronics. Nonuniform strain is present or can even explicitly be applied in experiments in the form of wrinkles, folds, or when placing 2D materials on prepatterned surfaces.^14–17^ Wrinkles are particularly interesting as they can unintentionally or intentionally be formed on 2D materials.^18–22^ They can also be used as a model system for nonuniformly strained systems such as bubbles or atomic-scale protrusions.^23^ Although periodically wrinkled structures under compression can be different from delaminated wrinkles that form in TMDC layers transferred onto a substrate,^24^ the strain fields still exhibit similar complex features, such that most of the physics will be comparable.^25^ Furthermore, in experiments, effects due to the moiré potential in heterostructures and due to wrinkling can both lead to similar excitonic effects,^26–29^ and the investigation of wrinkled structure thus helps to distinguish them.

2D materials also offer a unique platform for fundamental studies of neutral and charged excitons.^30,31^ Among the class of 2D materials, TMDCs are considered as an optimal candidate for optoelectronic investigations and applications due to their unique electronic properties, such as the spin-valley optical selection rules^32^ and different excitonic species.^33,34^ In this publication, we use X^eh^ for the excitons having electron and hole on the same layer and IX^eh^ for excitons with electron and hole states on different layers and where the superscripts indicate the momentum space location of the electron and hole, respectively. TMDC bilayers have their conduction band minimum (CBM) and valence band maximum (VBM) at different points in the Brillouin zone, such as the *Q* and *K* points in WSe_2_. This leads to momentum-indirect, interlayer IX^*QK*^ excitons^34^ since the CBM is partially delocalized on both layers.^35,36^ There is, furthermore, the momentum-direct intralayer exciton X^*KK*^ at the *K* point of the Brillouin zone, which is known from monolayer systems. Strain can modify the band structure and cause an indirect-to-direct band gap transition,^37^ but uniform strain will not change the character of the corresponding excitons at the *K* point. Momentum-direct interlayer excitons have been observed for MoS_2_ ^38^ and MoSe_2_;^39^ however, similar momentum-direct interlayer excitons have not yet been observed for WSe_2_. An overview of publications on interlayer excitons in TMDC homobilayers is presented in Table 1.

**Table 1:** Overview of Interlayer Excitons in Homobilayers of 2D TMDCs, e^–^ and h^+^ Represent Electrons and Holes, Respectively^a^

exciton type	momentum	material	references	localization	symbol
interlayer	momentum-direct	MoS_2_	^38^		
		MoSe_2_	^39^		
interlayer	momentum-indirect	WSe_2_	^34,44–48^	e^–^ at *Q*, h^+^ at *K*	IX^*QK*^
interlayer	momentum-indirect	MoSe_2_	^36^	e^–^ at *K*, h^+^ at Γ	IX^*K*Γ^
interlayer	momentum-indirect	WSe_2_	^34,48^	e^–^ at *K*, h^+^ at Γ	IX^*K*Γ^
interlayer	momentum-indirect	WSe_2_	^48^	e^–^ at *Q*, *h* ^+^ at Γ	IX^*Q*Γ^
interlayer	hybridized e^–^/h^+^	MoS_2_	^49^	hybridized h^+^	
interlayer	hybridized e^–^/h^+^	MoS_2_	^49–51^	hybridized with B exciton	

a Hybridized excitons have both momentum-direct and indirect characters. The corresponding symbol used in this publication is added to the last column.

TMDC heterostructures have also attracted significant attention due to their type-II band alignment (i.e., the VBM and CBM are localized on different layers) and the resulting formation of interlayer excitons.^40–42^ Interlayer excitons can be employed more effectively in devices since they have longer lifetimes due to the reduced overlap of electron and hole wave functions.^43^

In this investigation, we show how the wrinkling of homo- and heterobilayers of 2D TMDCs leads to the localization of the band edges and the reduction of the local band gap. On the basis of our single-particle calculations, we can infer that nonuniform strain can induce momentum-direct interlayer excitons, IX^*KK*^, in homobilayers.

## Results

### WSe_2_ Bilayer Wrinkle

We create the wrinkled WSe_2_ homobilayers by compressing a relaxed flat 2H homobilayer of a 1 × 15 × 1 rectangular WSe_2_ unit cell (i.e., reducing the size of the cell in the armchair direction). Due to band folding, we have chosen the direction leading toward the armchair termination (please refer to Methods section for more details). In this publication, we use the term “compression” for the reduction in the lattice parameter and “strain” for the local strain arising from changes in atomic distances. The resulting wrinkles (please see Figure 1a) lead to several variations in the system’s electronic structure. However, to understand the origin of such changes, it is important to first investigate the structural changes themselves.

**Figure 1:**
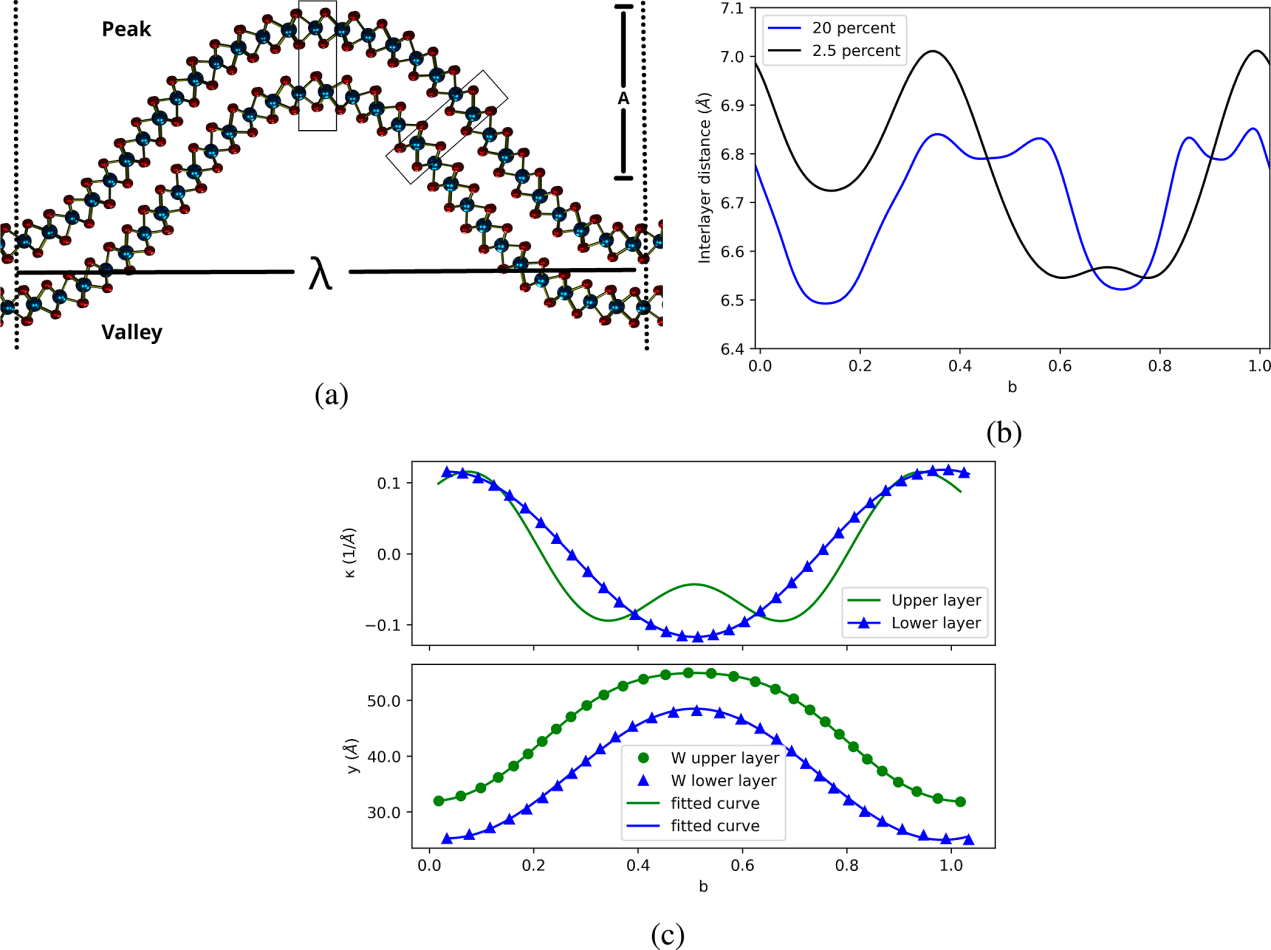
Structural changes of the homobilayer WSe_2_ under wrinkling. Nonuniform strain is complex and varies bond lengths, stacking, and interlayer distance. (a) The wrinkled homobilayer structure WSe_2_ with 20% compression, two exemplary areas with different stacking are indicated by rectangles. (b) Interlayer distance of the WSe_2_ homobilayer wrinkle at two different strain values of 2.5% and 20%. (c) (upper panel) Curvature, κ, and the atomic positions of the W atoms of wrinkled WSe_2_ (lower panel) for 20% compression—further details on the fit function can be found in the Supporting Information. *b* is the unit vector of the lattice parameter in the direction of the wrinkle.

### Atomic Structure

The relaxed lattice parameter of homobilayer WSe_2_ is 3.294 Å, which is in good agreement with the literature.^52–54^ The interlayer distance of 6.575 Å is also in an acceptable agreement with the 6.435 Å and 6.50 Å found in refs 52 and 54 , respectively. After relaxation of the bilayer, it is compressed, and the atoms relax in the out-of-plane direction, forming the wrinkled structure. The resulting nonuniform strain is complex and involves fluctuations in the W–Se bond lengths, which depend strongly on the chalcogen position. However, the reported maximum strain values refer primarily to changes in the W–W distance. For example, in the relaxed flat homobilayer, the W–W distance equals 3.294 Å, and at 2.5% compression, we have max­(W–W) = 3.313 Å and min­(W–W) = 3.286 Å, which correspond to a strain of 0.5% and −0.2%, respectively. Figure 2 depicts the structures for three exemplary compressions and the strain distribution in these structures. There is a nonmonotonic variation in the maximum and minimum strains, which is probably due to small local slip of the layers and local stacking changes (please refer to Table S1 for more detail on the local strain values in the structures). Most interestingly, while the wrinkle formation leads to compressive and tensile strain for the inner and outer W–Se bonds in each curve, the W–W bonds experience a stronger tensile strain.

**Figure 2:**
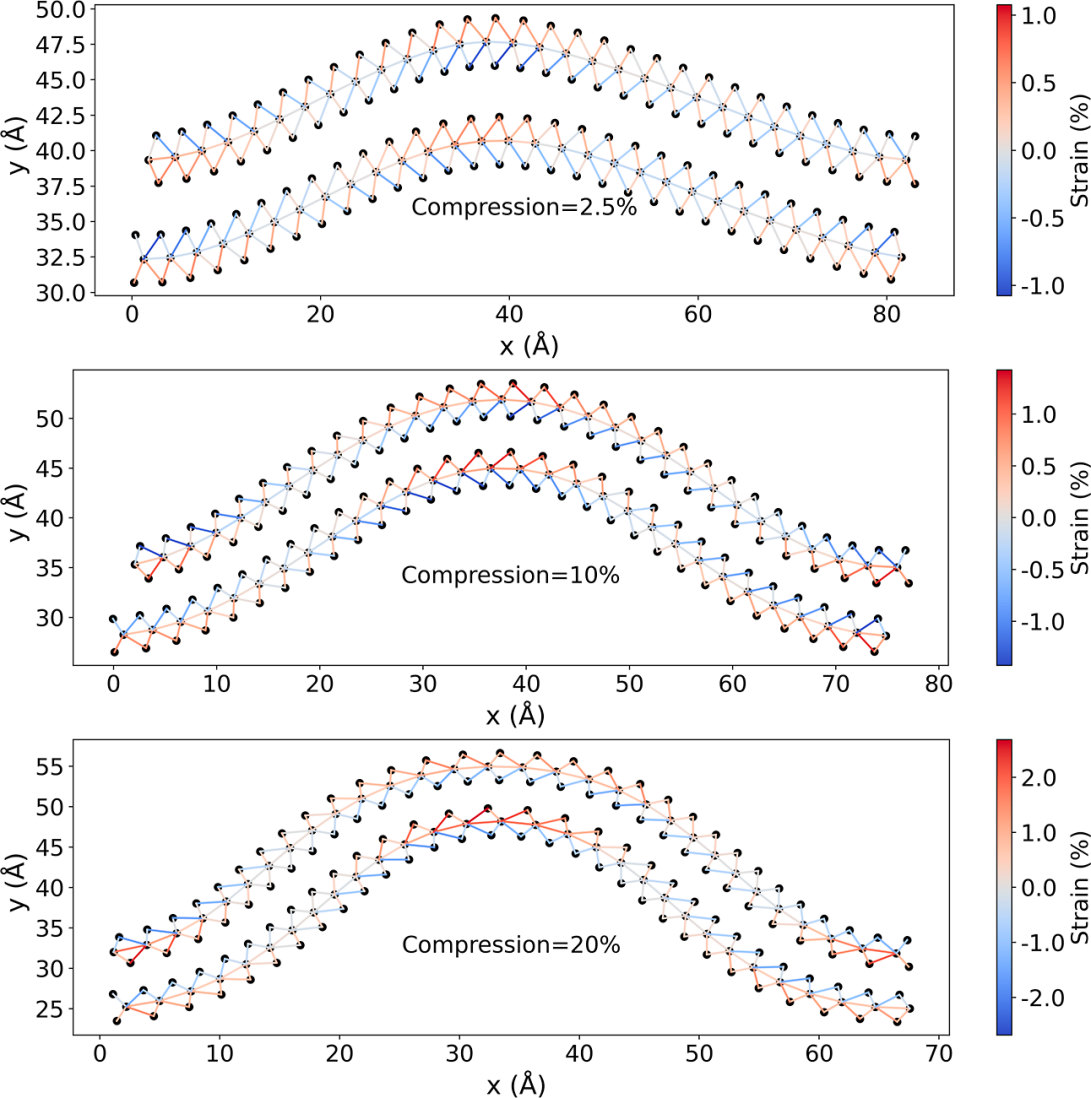
Strain variation along the homobilayer WSe_2_ wrinkle for three exemplary compressions of 2.5%, 10%, and 20%. The structures not only have a variation of strain in the wrinkle direction but also on each side of the metal atoms. Bond strain for W–W and W–Se bonds is defined as $\varepsilon =\frac{d-d_{flat}}{d_{flat}}\times 100\%$ , where *d* and *d*_flat_ are the distances between atoms in the strained and flat, unstrained system, respectively. n.b. the range of strains value is different in each figure. Please refer to Figure S2 in the Supporting Information for the other compressions.

The nonuniform strain effects in different spatial positions of monolayer TMDCs have been discussed in several theoretical and experimental studies.^25,55,56^ However, its effects on bilayers have not been thoroughly investigated. Figure 1a shows the relaxed structure of homobilayer WSe_2_ after 20% compression. It obtains higher strain at the peaks and valleys of the structure and retains a lower strain in the connecting area. Also, at the peak (valley), the upper layer has lower (higher) strain than the lower layer. Moreover, the bilayers have two extra degrees of freedom. They not only relax in the out-of-plane direction, but also their interlayer distances, as well as their stacking, are affected. These two points are not considered in previous studies to the best of our knowledge. Figure 1b depicts the minimum distance between the splines passing through the positions of the metal atoms on different layers. The distance between these two splines fluctuates between two values. The minimum distance between the splines in the wrinkled structures is close to that in a flat homobilayer of WSe_2_, while the maximum distance can be up to 0.5 Å larger. At a lower compression of 2.5%, the two layers have larger flat areas, and the curved regions are smaller. On the other hand, at 20% compression, the flat areas are smaller, and the curved regions are larger. Moreover, peak and valley positions are flatter (Figure 1c). As shown in Figure 1a, the bilayer stacking is changed. It does not correspond to the 2H stacking any longer (*H*_h_^h^ in the nomenclature of ref 57). There are sections in the structure where the chalcogen and metal atoms are not above each other, and the stacking is closer to, e.g., *H*_h_^W^. The layers additionally have different curvatures along the wrinkles. There is a small variation of curvature in the vicinity of the peaks. We speculate that small sliding between layers and changes in stacking are the culprits of this behavior. In this way, each layer’s strain state differs at the highest curvature sections (valleys and peaks). These structural variations and the localization of curvature alter the electronic structure, as we discuss in the following section.

### Electronic Structure

The flat homobilayer WSe_2_ is an indirect-band gap semiconductor with its valence-band maximum (VBM) at the *K* and its conduction-band minimum (CBM) at the *Q* points of the reciprocal space (see Figure S1). In our calculation, its indirect band gap is 1.159 eV (without spin–orbit coupling (SOC) 1.382 eV), and the difference between the indirect and direct band gaps is about 174 meV. These values are in good agreement with previous investigations.^53,58,59^

A flat 1 × 15 × 1 rectangular supercell of the system has the same dispersion in the Γ–*X* direction as the rectangular unit cell, but with 15 times degenerate bands. The wrinkling breaks the translational symmetry along the wrinkle (the *x*-axis in Figure 2) by moving atoms in the out-of-plane direction, thus leading to band splitting. Figure 3a shows the band structures of the wrinkled WSe_2_ homobilayer at 20% compression along the Γ–*X* line to which the bands of the hexagonal unit cell are folded (please refer to Figure S3 for the other band structures at different compressions). With increasing strain, the CBM at the backfolded *K* point moves down, and the *Q* point moves up, leading to an indirect-to-direct transition^60^ (see also Figure S1). Due to the small difference between the direct and indirect band gaps of the unstrained WSe_2_ homobilayer, we find that the wrinkled structure undergoes an indirect-to-direct transition even for a small strain of 0.6% which occurs at 10% compression and above (Table S1). This can explain the increased photoluminescence observed in several experimental investigations.^37,60^ It is worth reiterating that the strain field is a complex phenomenon, and it is not possible to correlate the highest strain with band-edge shifts. However, to obtain an overview of the strain values in the structure, it can be useful to compare the maximum strain with the band gap variation along the wrinkle. For example, at 20% compression, the system exhibits maximum tensile and compressive strains of 1.87% and −2.3%, respectively, leading to band gaps of 1.094 to 1.281 eV at different locations along the wrinkle. For further information, please see Figures S1 and S4 in the Supporting Information.

**Figure 3:**
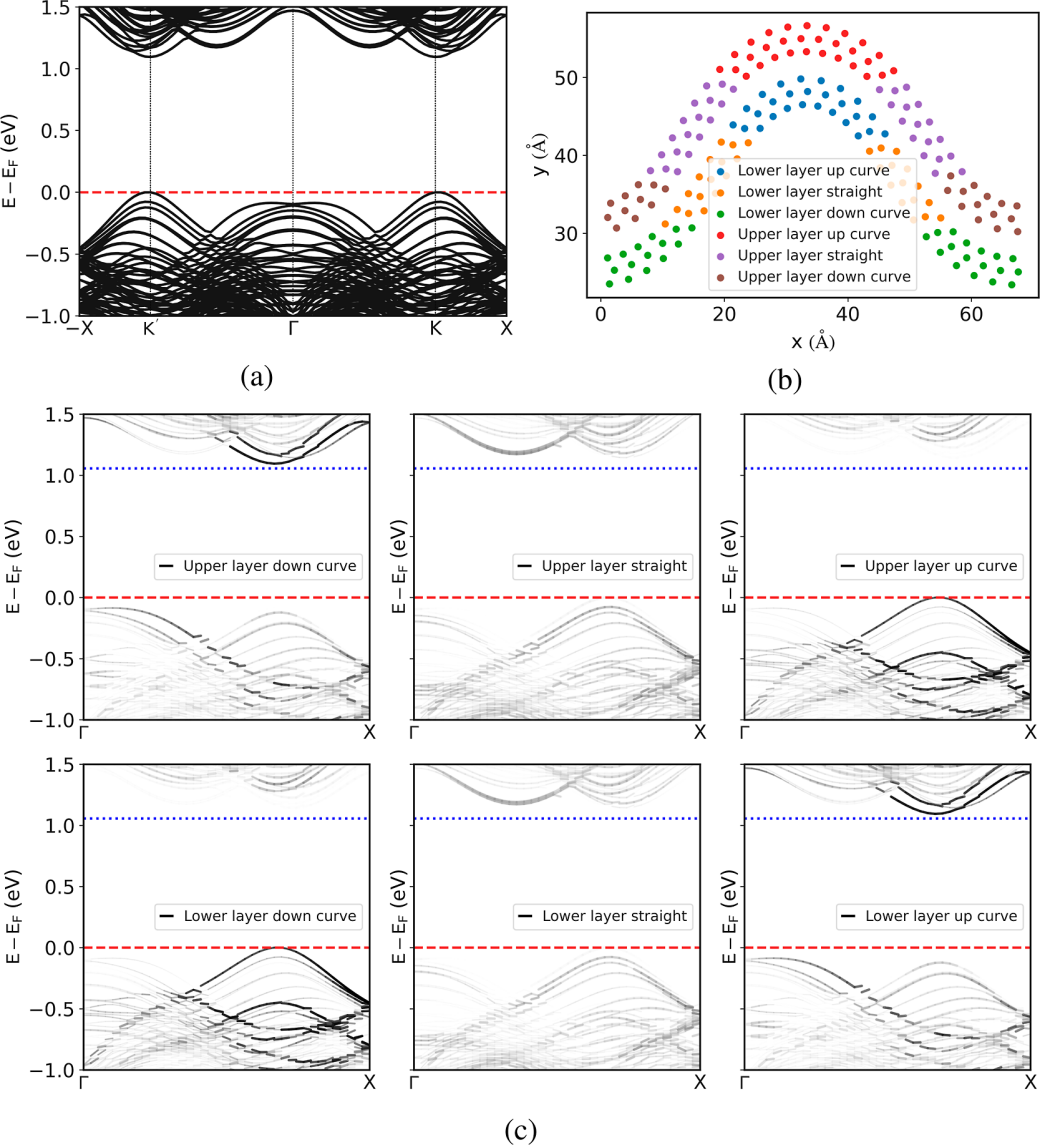
Localization of states in different regions and layers of the wrinkled homobilayer. (a) Band structures of the WSe_2_ homobilayer at 20% compression, (c) contribution of states from different atoms to the band structure of the wrinkle as sketched in (b). The color intensity indicates the amount of the contribution from that section. The solid black line indicates that all the states are localized in the specified region. The energy values are shifted relative to the Fermi energy, *E*_F_.

Figure 3 shows the band structure and the contribution of different sections of the wrinkles to the bands of the 20%-compressed bilayer WSe_2_. In Figure 3c, the intensity of a line indicates the contribution to a band by states localized in a specific section. Hence, a solid black line would indicate that the band is formed only by states that originate from that section. We concentrate here on the results for the 20%-compressed system, as it provides a clearer picture due to the larger strain in the system. Band structures for all other compressions are provided in the Supporting Information. Due to the (approximate) inversion center at the inflection point of the wrinkle, CBM and VBM are both formed by two energy-degenerate bands, which are localized in either of the two curved regions. Focusing on the curved area at ≈0.5*b* (see Figure 1c), the CBM is mostly contributed from the states located on the lower layer, and the VBM is more localized on the upper layer. These different localizations can be related to the curvature-induced dipoles^25,61,62^ —the curvature-induced flexoelectricity leads for TMDCs to a downshift of both band edges for moderate curvatures.^62^ Since the curvature is larger at the inside of a curve, the band edges are lower in energy at the inside than at the outside. For 20% compression, almost 80% of the valence band (VBM – 1) is localized on the upper layer, and more than 90% of the conduction band (CBM) is localized on the lower layer. Therefore, the electrons and the holes of the excited electron–hole pairs have a higher probability of being localized in the lower and upper layers, respectively. We want to emphasize that this behavior is very different from the calculations for uniformly strained systems, which attribute the localization of excitons solely on the reduction of the band gap^19^—here, the nonuniform strain leads to a localization of the band edges in different high-strain areas of WSe_2_. Table S3 shows the quantitative values of the contribution to the band edges for different sections of the 20% compressed wrinkle (the sections are indicated in Figure 3).

The nonuniform strain thus effectively leads not only to the localization of the states and may result in a funneling of the excitons to the strained regions but also to the formation of interlayer excitons in the WSe_2_ homobilayer. This separation of the electron and hole wave functions on different layers is significant, as it can lead to a longer lifetime of the excitons. Yet, in contrast to the case of heterobilayers, the energy difference between the inter- and intralayer excitons is here only determined by the difference in the binding energy. The binding energy for the intralayer exciton in the wrinkle will furthermore reduce since the electron and hole are localized in different regions of the system, as can be seen in Figure 3. To provide a better spatial view of the states, the eigenstate density, |ψ|^2^, of the states corresponding to the band edges VB – 1, VB, CM, and CM + 1 of 20% wrinkle is shown in Figure 4 with electron- and hole-like states indicated by blue and red colors, respectively. Table S4 additionally summarizes the local band gaps in different sections of the homobilayer at different compressions.

**Figure 4:**
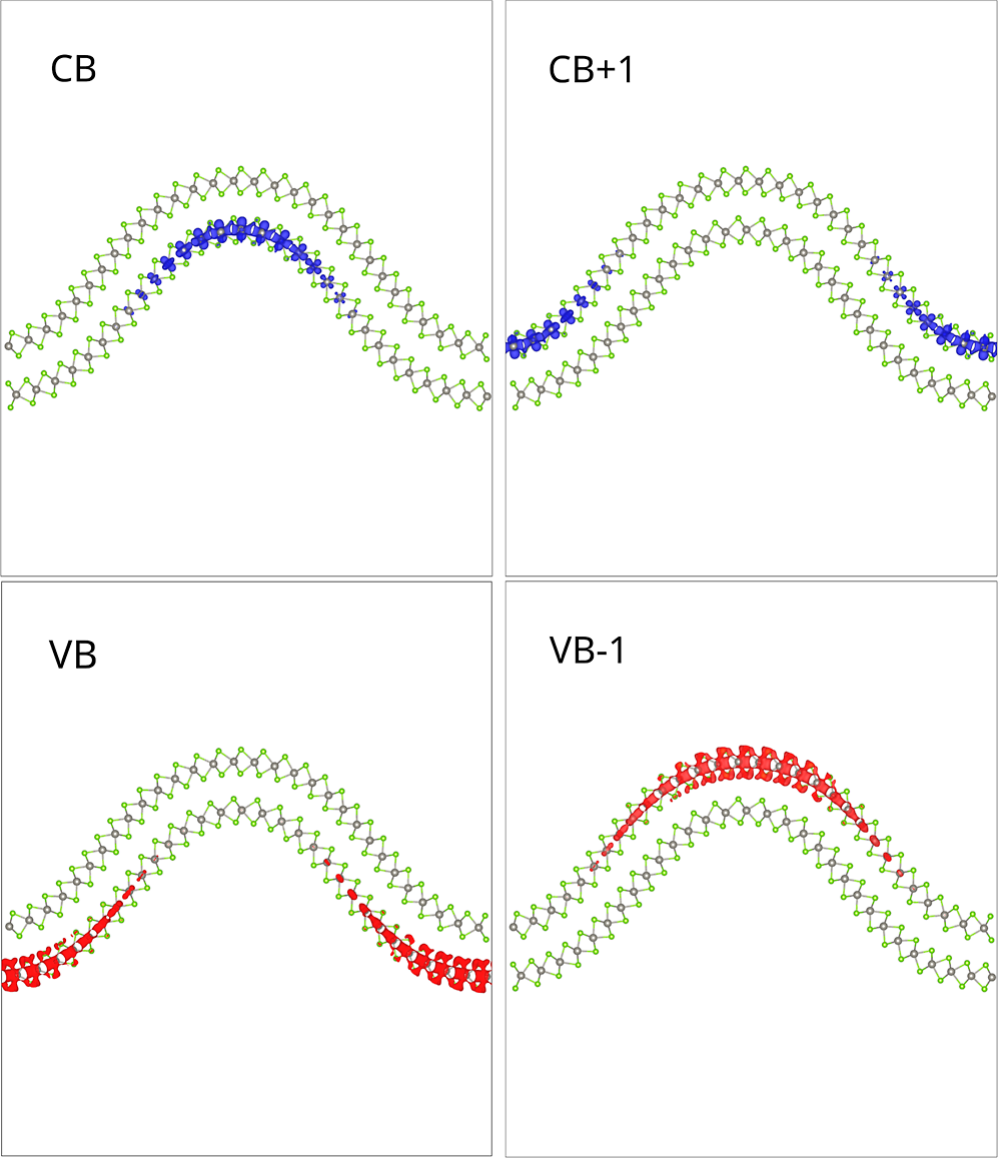
Electrons and holes are localized at different spatial positions of the bilayer wrinkle, leading to the formation of interlayer excitons in homobilayer WSe_2_. The VB – 1, VB, CB, and CB + 1 eigenstate density |ψ|^2^ is depicted to show electron localization in blue (up) and hole localization in red (below) for the 20% compressed homobilayer WSe_2_.

For small strain, the backfolded bands of the *Q* point (which are still lower in energy) hide the CBM at *K*. Additionally, the CBM and VBM are degenerate states; however, plotting the spin textures of the VB – 1, VB, CB, CB + 1 individually (see Figure S19) unravels that the smallest direct gap at *K* which is dark in monolayer WSe_2_ (spin forbidden) becomes brighter after compression. It should be noted that this brightening is different from the brightening of the excitons due to the mixing with defect states^63^ and that it is directly linked to the formation of the interlayer exciton IX—the spin-layer locking^64^ leads for tungsten-based TMDCs in the 2H-stacking to a spin-allowed transition if the electron and hole are localized on different layers rather than on the same layer. For further details, please, see section Spin Texture in WSe_2_ Bilayer Wrinkles in the Supporting Information.

Furthermore, the VBs near the Γ point show a Rashba-like splitting due to the curvature-induced electric field (see Figure S3j for a magnified view), which is, however, smaller (α_*R*,max_ = 0.1151 eV Å for the wrinkle at the 20% compression) than the splitting in the wrinkled TMDC monolayer.^25^ The Rashba parameter is also 1 order of magnitude smaller in bilayer TMDCs than in flat monolayers under an external electric field.^65^ This might be due to the reduction of the out-of-plane dipole moments and the curvature (Table S2) at the outer surface.

The change of the metal atoms in one of the layers of a bilayer alter the system’s properties by not only breaking the inversion symmetry—TMDC heterobilayers are particularly interesting because they can host interlayer excitons due to their type-II band alignment. Therefore, in the next section, we consider the wrinkling of WSe_2_MoSe_2_ heterobilayers and investigate alterations in the electronic structure due to nonuniform strain.

### WSe_2_MoSe_2_ Heterobilayer Wrinkle

The heterobilayer is constructed by replacing W in the lower layer with Mo atoms. The heterobilayer wrinkles are then formed by the same procedure as that for the homobilayers. In the following section, we first discuss the geometrical variation in the structures due to the wrinkling, and in the subsequent section, we explain how this changes the electronic structure.

### Atomic Structure

The heterobilayer relaxation is quite similar to the homobilayer WSe_2_. The peaks with different strain states in each layer are connected by areas of lower strain. Yet, due to the different bending stiffness of MoSe_2_ and WSe_2_, the structure (and thus the local strain) does not have an inversion center. The strain is of a complex nature in these structures, as the layers also adjust the interlayer distance and the stacking. The interlayer distance is 6.555 Å for the flat WSe_2_MoSe_2_ heterobilayer. Figure 5 demonstrates the variation of the distance between the metal atoms of the heterobilayer WSe_2_MoSe_2_ in the two cases of 2.5% and 20% compressions. The distance between the layers is fluctuating along the wrinkle. Interestingly, at lower strain (i.e., lower compression), the variance of the interlayer distance is larger than in the cases with higher compression. Moreover, a similar stacking variation as in the homobilayer occurs in the heterobilayer. The strain in our freestanding heterobilayers (i.e., without a substrate) is generally smaller (≈2% at 20% compression) than in monolayer systems (≈3% at 20% compression) such as MoSe_2_ ^66^ due to the higher bending stiffness of the heterobilayer. Table S11 summarizes the maximum and minimum strain in each layer of the heterobilayer. The interlayer distance and stacking profoundly influence the electronic structure; hence, in the next section, we explain the changes in the electronic structure induced by wrinkling.

**Figure 5:**
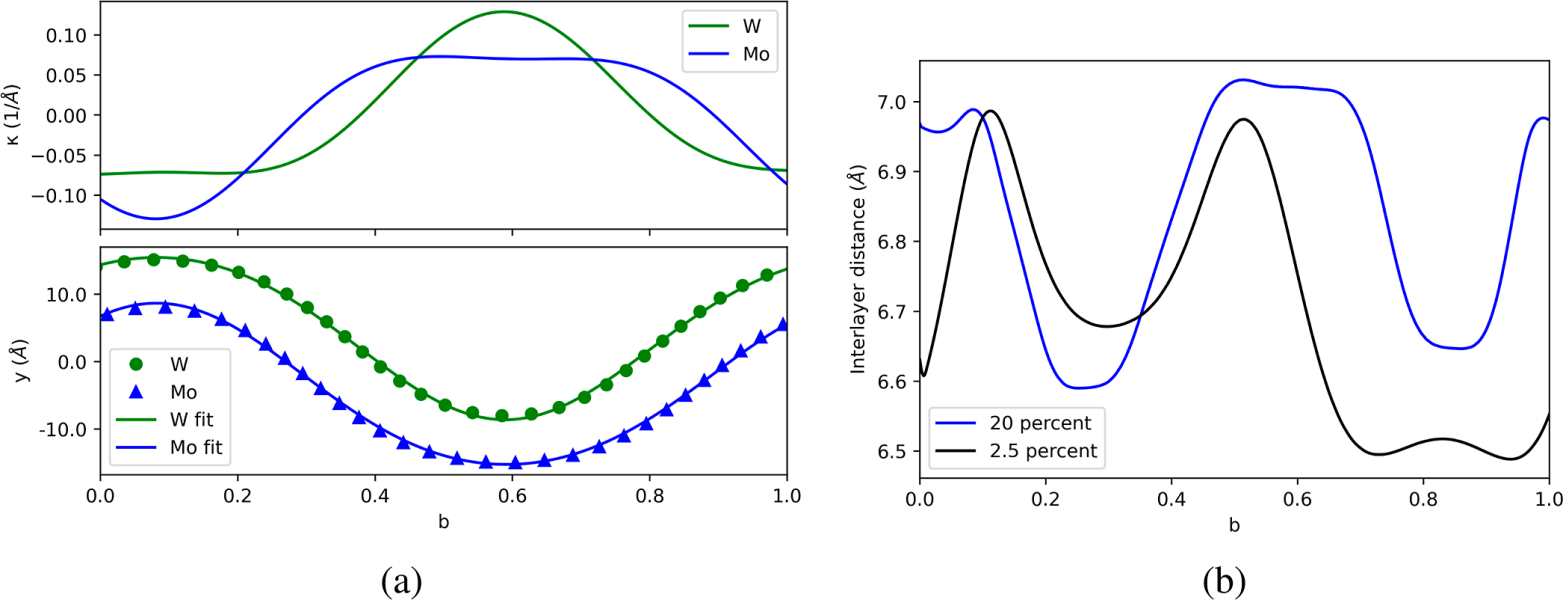
Structural changes due to wrinkling of the heterobilayer WSe_2_MoSe_2_. The layers have different local strain and stacking, as well as a variation of the interlayer distance along the wrinkle direction. (a) (upper panel) Curvature, κ, of the fit function of the metal atoms’ position of wrinkled WSe_2_MoSe_2_ (lower panel) with a compression of 20%. (b) Interlayer distance of the WSe_2_MoSe_2_ heterobilayer wrinkle at two different compression values of 2.5% and 20%—please note that the wrinkles have a different wavelength and the distance is shown w.r.t. the fixed cell axis.

### Electronic Structure

The flat heterobilayer WSe_2_MoSe_2_ is also an indirect band gap semiconductor (band gap equals 1.005 and 1.229 eV with and without SOC, respectively, cf. Figure S1) with the VBM and CBM localized at the *K* and *Q* point, respectively (experimental indirect band gap 1.31 eV).^67^ The difference between the direct and indirect band gaps (76 meV) is smaller than in the case of the homobilayer WSe_2_ (174 meV). Having an indirect band gap close to the bright exciton energy can lead to nonradiative losses in devices based on this material.^60^ Additionally, in WSe_2_MoSe_2_ heterobilayers, the CB and VB are localized on different layers due to the type-II band alignment.^68,69^ This leads to an increased lifetime of the corresponding interlayer excitons in comparison to the intralayer excitons.^70^ Moreover, heterostructures have an intrinsic electric dipole that changes their band structures^59^—it can, for example, lift the degeneracy of states as well as couple with the intrinsic spin momentum of the system. Figure 6a shows the band structure of the wrinkled structure of the heterobilayer at 20% compression, and Figure S22 summarizes all the band structures of the WSe_2_MoSe_2_ heterobilayer for the compressions studied. At 2.5% compression, the band structure remains very similar to that of the flat system. At this compression, the maximum strain in the structure is 0.6% (cf. Table S11). VBM and CBM occur at the backfolded *K* point; however, the difference between the direct band gap and indirect band gap (at the backfolded *Q* point) is only 13 meV. By the increase in strain in the system, it goes through an indirect to direct transition, which can lead to a stronger photoluminescence of the system. Moreover, the direct band gap reduces from 1.082 to 0.931 eV.

**Figure 6:**
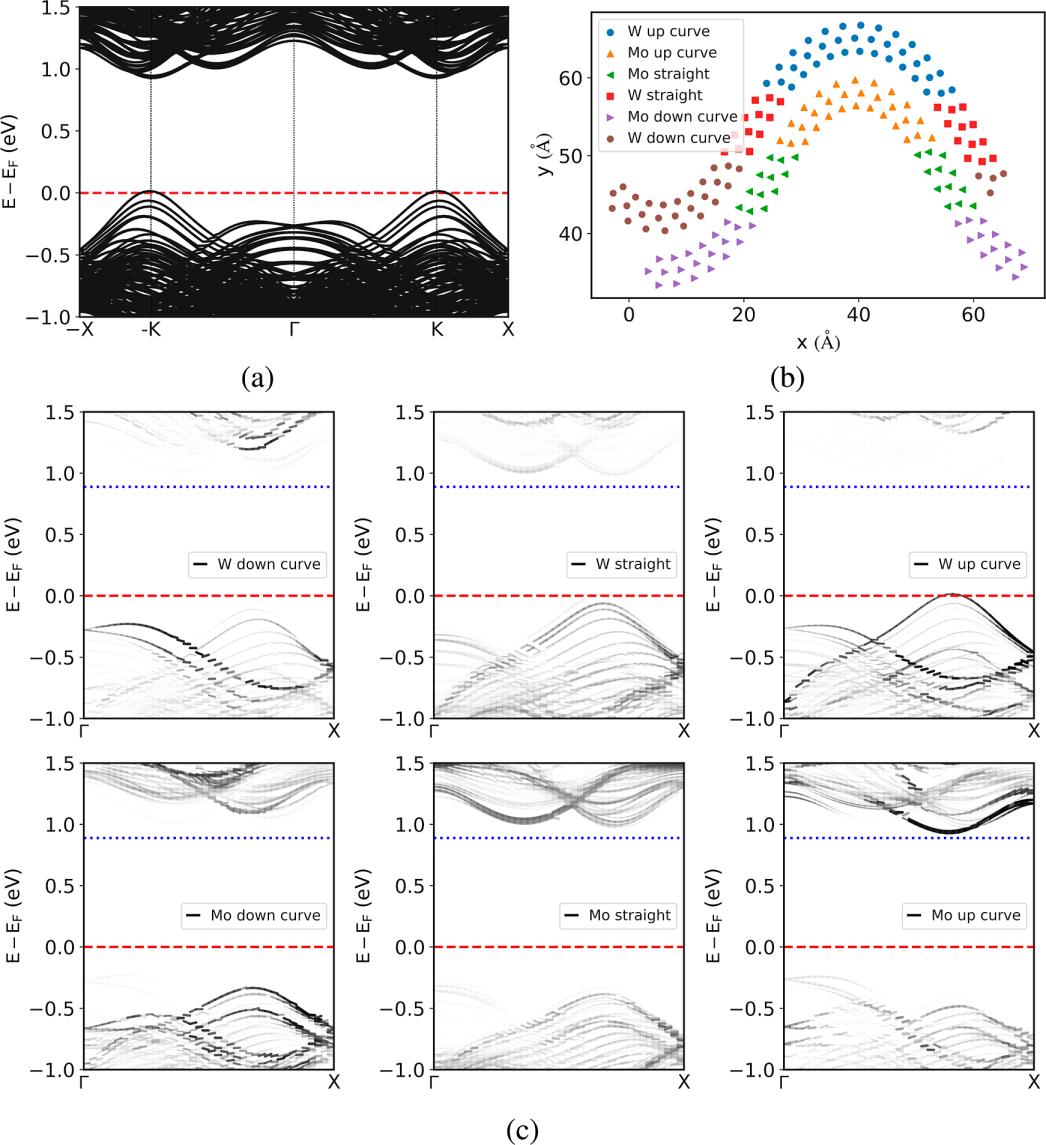
Localization of the states in different regions and layers of the wrinkled heterostructure. Band structure of the WSe_2_MoSe_2_ heterobilayer at 20% compression and (c) the contribution of the metal atom at different sections of the wrinkle, the location of VBM (red) and CBM (blue) are also indicated to help the eye; additionally, the position of contributing atoms to the band projection is given in (b).

Although the compressive strain eventually leads to a direct band gap,^53^ the out-of-plane relaxation reduces the strain present in the system. For example, in Figure S23 of the Supporting Information, the 5% compressed system with and without wrinkle formation is shown. The wrinkled system still has an indirect band gap in contrast to the direct band gap of the uniaxially strained system. The reduction of strain by out-of-plane relaxation is one of the driving mechanisms of the wrinkling in the first place.

Real space information on the band edges is obtained by projection of the states on spatial locations of the wrinkles. Figure 6 shows the band structure and its projected band structure on different sections of wrinkled WSe_2_MoSe_2_ at 20% compression, which, due to higher local strains, improves the depiction of changes that are also present in lower compressions. It can be inferred that the VBM is mostly made of W states in the upper curved area, whereas the CBM originates from the Mo states in this section. This is particularly important as the spatial separation of electrons and holes can enhance the lifetime of the excitons (IX^*KK*^), which will be relevant for the effective employment of these structures in electronic devices. Similar to the homobilayer, the band edges’ eigenstate density |ψ|^2^ for VB – 1, VB, CM, and CM + 1 states of the 20% compressed wrinkle are shown in Figure 7. In this figure, the holes (electrons) are shown in red (blue). For reference, Figure S24 shows the projected bands on different sections at 2.5% compression. Strain dependence of the localization is also apparent from Table S12, which summarizes the band gap at different sections of the heterobilayers for different compressions.

**Figure 7:**
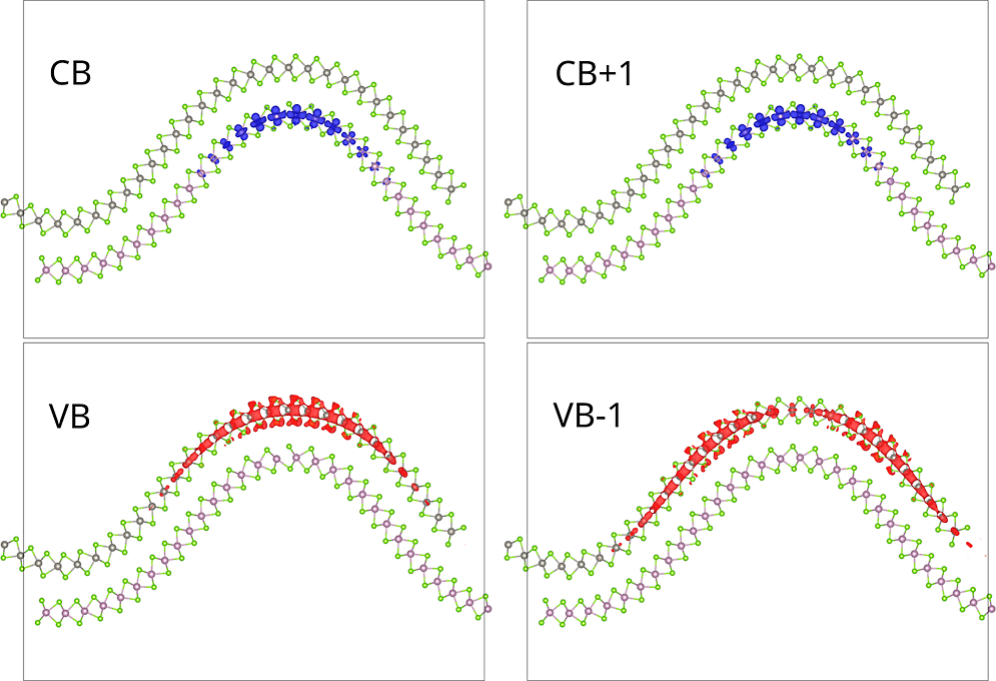
Electrons and holes are localized in the curved area of the heterobilayer wrinkle, in which the Mo layer has a higher curvature. The VB – 1, VB, CB, and CB + 1 eigenstate density |ψ|^2^ is depicted to show electron localization in blue (up) and hole localization in red (below) for the 20% compressed homobilayer WSe_2_.

The expectation value of the Pauli matrices reveals connections between the wrinkling and mixing of the spins. At the backfolded *K* point, the CB and CB + 1 states become spin-mixed. Because of the conduction band and valence band possessing different orbital magnetic quantum numbers, *m*_l_, the allowed excitations in TMDC monolayers are those where the states have the same spin state.^32^ The spin mixing we find in the wrinkled heterobilayers can thus be the reason why some researchers observed the dark exciton states in experiments.^63^ Furthermore, the curvature will lead to a mixing of in-plane with out-of-plane states similar to the case of carbon nanotubes.^71^ This mixing of states with different *m*_l_ values will also influence the optical selection rules and thus can change the photoluminescence at the strained regions at the peaks. For a general overview of the expectation values of the Pauli matrices for different wrinkles, as well as the *z* component of the expectation value of Pauli matrices at various strain states, please refer to the section Spin Texture in WSe_2_/MoSe_2_ Heterobilayer Wrinkles in the Supporting Information.

Heterobilayers have an intrinsic potential gradient^65^ that breaks the symmetry of the system and leads to momentum-direction splitting (i.e., Rashba-like splitting).^72^ As can be inferred from Figure S22, the splitting increases with strain. Unfortunately, unlike for wrinkled TMDC monolayers,^25^ we have not observed any case with the splitting in the heterostructure to occur above the VBM energy at *K*. In this way, it is difficult to observe this splitting in experiments. Nevertheless, the presence of such a splitting is significant, as it can be further modified by external electric fields^73^ or proximity effects.^74^

In order to disentangle how the curvature and the interaction between the layers affect the Rashba-like splitting, we also calculated the electronic structure of separated wrinkled layers by keeping their shape profiles. Looking at Figure S30 in the Supporting Information, the Rashba-like splitting of each layer is larger than that in the combined structure. Hence, we conclude that the interaction of the two layers reduces the Rashba-like splitting in the TMDC heterostructures, which may be due to the screening effects.

### Stacking-Induced Changes and Sulfur-Based TMDCs

Until now, we have investigated the standard 2H stacking of homo- and heterobilayers as the initial structure. Yet, in experiments, the sample preparation, especially for heterobilayers, can lead to (or can be used to create) twisted structures in which different stacking sequences are realized. In order to understand the robustness of our predictions on the formation of different exciton species in such situations, we also included the *H*_h_^X^ and *R*_h_^h^ stackings in our homobilayer calculations. In flat bilayers with the *H*_h_^X^ stacking, the second layer is rotated with respect to the first by 60° similar to the *H*_h_^h^ stacking. In contrast to the *H*_h_^h^ stacking, however, the metal atoms are now aligned.^75^ When the *H*_h_^X^-stacked bilayers are compressed, the layers initially slide over each other and the system changes to the *H*_h_^h^ ground state before wrinkling—due to the weak van der Waals bonding between the layers, even a weak external perturbation, such as a small inhomogeneous strain is enough to trigger this transition.

In contrast to 2H stacking, in *R*_h_^h^ stacking, the two layers do not exhibit a twist angle. Compressing the *R*-stacked homobilayer leads to very similar structural changes and the band-edge states also localize on the peaks (i.e., high-strain regions) of the wrinkle. One important difference, however, is the order of the spin states.^76,77^ In tungsten-based monolayer TMDCs, the lowest–energy transition is spin forbidden and thus dark. Since the 60° rotation in 2H-stacked bilayers leads to a mapping of the *K*′ point of one layer to the *K* point of the other, the interlayer transition becomes spin allowed and thus bright. Yet, this is not the case anymore for *R*-stacked bilayers because the 0° rotation effectively results in the same spin of the band edges in each layer. Please see Figure S14 in the Supporting Information.

Additionally, we calculated the wrinkles of homobilayer MoSe_2_, which show similar behavior to that of homobilayer WSe_2_ (please see Figure S18 in the Supporting Information). Yet, because of the different spin orders of the band edges in Mo-based TMDCs, the lowest interlayer transition in 2H stacking is optically dark, while it is bright for *R* stacking.

Finally, we also calculated the WS_2_MoS_2_ heterobilayer, which behaves very similarly to the WSe_2_MoSe_2_ case—the band edges localize in the area with the largest strain in the MoS_2_ layer. Further details can be found in the Supporting Information.

## Conclusions

Although uniform strain is known as a tool to modify the exciton dynamics in TMDCs, the role of nonuniform strain on excitons is more elusive. We have shown here that nonuniform strain can lead to unique localization of the band-edge states in bilayer TMDCs. Unfortunately, the ab initio calculation of excitons in our wrinkled systems is (currently) not possible due to the large size of the system and the presence of many bands close to the CBM and VBM. However, as a first approximation, the exciton’s binding energy is proportional to the effective masses of the electron and hole and inversely proportional to the in-plane polarizability.^78^ The latter, in turn, is inversely proportional to the band gap. The effective mass variation in, e.g., the WSe_2_ homobilayer is quite small—the electron and hole masses are changing during the wrinkling by approximately 7% and 1%, respectively. Hence, the reduced mass of excitons varies less than 4.5%, resulting in a variation of the exciton energy by a few tens of meV at most. On the other hand, the band edges are shifting by as much as 100 to 200 meV. Therefore, the changes of the band edges have a stronger effect on the absolute exciton energy. These changes and the band-edge localization can result in rather unexpected properties of the excitons in the homo- and heterobilayers, which can be summarized as follows:

- localization of the states due to nonuniform strain could lead to the formation of localized, momentum-direct interlayer excitons, IX^*KK*^, in homobilayer WSe_2_.
- interlayer excitons, IX^*KK*^, in wrinkled/inhomogeneously strained homobilayers, localize in high-strain regions
- intralayer excitons, X_A_, in wrinkled/inhomogeneously strained homobilayers will have a lower oscillator strength^79^ as compared to monolayer systems due to the electron and hole being localized in different valleys of the wrinkle.^80,81^

- interlayer excitons in wrinkled/inhomogeneously strained heterobilayers localize in the area with the largest strain in the Mo layer.
- intralayer excitons, X^*KK*^, will also have lower oscillator strength as compared to monolayers due to the larger electron–hole separation^80,81^

The reduction of the oscillator strength in comparison to the monolayers and flat bilayers is confirmed by calculations of the momentum-matrix elements (MMEs), as shown in the Supporting Information. The MMEs for the intralayer, spin-conserving transitions are reduced by at least 1 order of magnitude. We associated this behavior with the localization of the initial and final states in different curves of the wrinkle. Furthermore, some MMEs for out-of-plane polarized light are increased considerably due to the wrinkle formation since this essentially mixes the in- and out-of-plane directions.

All the properties just mentioned above will influence the exciton diffusion in bilayer systems, and especially, the increased diffusion due to the excitonic dipoles should be measurable in pump–probe and time-resolved photoluminescence experiments.^82,83^ In homobilayers, inhomogeneous strain can lead to an increased photoluminescence due to the formation of momentum-direct interlayer excitons, IX^*KK*^, and due to the indirect–direct band gap transition. Furthermore, the interlayer excitons, IX^*KK*^, which will form, have an out-of-plane dipole in contrast to the intralayer excitons. Thus, they will repel each other, leading to increased diffusion. However, also the intralayer excitons now have a preferred direction for the dipole moment, which is from one curved region to the other. This will again lead to a repulsion. For heterobilayers, the preferred formation of interlayer excitons in the high-strain areas of the Mo-based TMDC will lead to a higher exciton density in this region and accordingly to a larger diffusion. Intralayer excitons will again have a preferred orientation for the dipole moment. Yet, this will be opposite for MoSe_2_ and WSe_2_—where we find the electrons in WSe_2_, there are the holes in MoSe_2_ and vice versa. This will again lead to different behaviors for the intralayer excitons, which in turn will also influence the interlayer ones. We furthermore found that the inhomogeneous strain leads to a change in the spin expectation values of the band edges, indicating a brightening of formerly dark, spin-forbidden transitions. We would like to reiterate that the changes in the exciton binding energy are complex, and further ab initio calculations and exciton dynamics^84^ should be conducted. Even though the exciton behavior of the wrinkled TMDC bilayers was elucidated in this research, the strain fields in various systems, such as bubbles, pre-strained surfaces, and even folds due to pre-strained substrate, should have similar behaviors. These different types of substrates will also lead to important differences in the dielectric screening, which are, however, difficult to generalize. Yet, since the screening affects all types of excitons, one could imagine combining the exciton localization via varying dielectric environments^85,86^ with the inhomogeneous strain localization. It is worth mentioning that the strain in the experiment is usually a complex phenomenon, having both uniform and nonuniform characteristics.

This work is closely related to the strain-induced manipulation of excitons in interesting applications such as exciton transistors, single quantum emitters, or exciton interconnects.^87^ For example, one may employ exciton funneling and leverage the brightness of the excitonic state, combined with the material’s strain sensitivity, to represent the on and off states.

## Methods

The wrinkles of homobilayer WSe_2_ and heterobilayer WSe_2_MoSe_2_ have been investigated by means of density functional theory as implemented in the all-electron code FHI-aims.^88^ The Perdew–Burke–Ernzerhof exchange correlation functional,^89^ Tkatchenko-Scheffler dispersion correction,^90^ and a nonself-consistent SOC^91^ have been utilized to calculate the electronic structure of the systems. The initial structures were created with a 1 × 15 × 1 supercell of the relaxed rectangular unit cell of bulk-like stacked 2H^92^ homo- or heterobilayers, with the longer in-plane supercell vector being along the armchair direction. The structures are also periodically perpendicular to the wrinkle direction. For the heterobilayer, we placed MoSe_2_ in the WSe_2_ lattice, which results in a minor mismatch of 0.15%. Subsequently, the flat structures have been fully relaxed to forces and pressures below 0.001 eV/Å and 0.1 bar, respectively. Then, the supercell was compressed along the armchair direction, and the atomic positions were relaxed again keeping the reduced lattice direction constant to retain the strain. The systems usually deform in the out-of-plane direction, and wrinkles are formed. However, it is worthwhile to mention that even with perturbing the positions of some atoms, few systems remained in the local minimum of a flat structure. Therefore, for these systems, we started from systems with larger compression which show the wrinkle formation and increased the lattice parameter again. Additionally, we performed calculations starting from a flat 2H homobilayer of WSe_2_ consisting of 1 × 10 × 1 WSe_2_ unit cells. These calculations confirm the results obtained using the larger unit cell presented in the main text and demonstrate that the results are not sensitive to the choice of initial conditions (see section Size Effect of Homobilayer WSe_2_ in the Supporting Information). Tables S2 and S13 summarize the structural parameters of wrinkled homo- and heterobilayer systems in this investigation. Mulliken band structures and spin textures were obtained from the final wrinkled structures. Mulliken band structure is calculated by summing the contributing coefficients of the basis on specific spacial locations, thus providing real-space information. We chose the armchair direction as the longer supercell axis due to band folding—compression and wrinkle formation along the zigzag direction fold many bands back to the Γ point, obscuring the identification of the valence band maximum. The interested reader might refer to the Supporting Information of ref 25 for further discussion.

## Supplementary Material



## Data Availability

The input and output files for the single-point calculations are publicly available in the NOMAD repository.^94^

## Notes

Fitting procedure, WSe_2_ bilayer wrinkle, WSe_2_ wrinkle in *R*_h_^h^ stacking, homobilayer MoSe_2_, spin texture in WSe_2_ bilayer wrinkle, WSe_2_MoSe_2_ heterobilayer wrinkle, spin texture in WSe_2_MoSe_2_ heterobilayer wrinkles, and heterobilayer WS_2_MoS_2_ (PDF)
