# Supplementary material for: Emergence of Nonuniform Strain-Induced Exciton Species in Bilayer Transition Metal Dichalcogenides
Source: ACS Nano. 2026 Jun 30;20(28):19930–41. doi: 10.1021/acsnano.5c09721 (PMC13394538; doi:10.1021/acsnano.5c09721)
Supplement: Supplementary file 1 [file nn5c09721_si_001.pdf]

Supplementary to emergence of non-uniform strain induced exciton species in bilayer transition metal dichalcogenides

Mohammadreza Daqiqshirazi[*,†,‡,¶] and Thomas Brumme[*,†]

†*Bergstrasse 66c, Theoretical chemistry, Technische Universität Dresden, Dresden, Germany.*

‡*Center for Advanced Systems Understanding (CASUS), Untermarkt 20, D-02826 Görlitz, Germany*

¶*Helmholtz Zentrum Dresden-Rossendorf, Bautzner Landstraße 400, D-01328 Dresden, Germany*

E-mail: m.daqiqshirazi@hzdr.de; thomas.brumme@tu-dresden.de

Supporting Information

Fitting Procedure

The location of metal atoms is fitted by a trigonometric function as follows:

$$f(x, a_0, a_1, a_2, a_3, a_4, a_5) = a_0 + a_1 \times sin(a_2 x) + a_3 \times sin(a_4 x + a_5) \tag{1}$$

Then, the first and second derivatives are calculated analytically, and the curvature is calculated by,

$$\kappa = \frac{y''}{(1 + y'^2)^{\frac{3}{2}}} \tag{2}$$

WSe_2 bilayer wrinkle

Figure S1 shows the atom projected band structure of the relaxed flat homobilayer WSe_2 and heterobilayer $WSe_2/MoSe_2$. Valence band maximum (VBM) and conduction band minimum (CBM) of the systems are located at K and Q point of their reciprocal lattice. In homobilayer WSe_2, the VBM is mostly formed from W states while the CBM has also contributions from chalcogen p states thus showing a clear interlayer character. Therefore, the exciton state originating from these reciprocal locations has both intralayer and interlayer character, and we refer to this as IX^{QK}. In the heterobilayer $WSe_2/MoSe_2$, the VBM is localized on the WSe_2 layer and the CBM is localized on the $MoSe_2$ layer, leading to a type II band alignment (i.e. the difference of the workfunction is smaller than the difference of the band gaps, hence electrons and holes can be delocalized on separate layers).[1,2]

The Strain variation in the wrinkle has a complex nature. Figure S2 shows the WSe_2 wrinkles with their strain variation for all the wrinkled systems in this study. The strain is compressive considering W−W distances, with higher strain at peaks; whereas the lower strain regions exist in connecting areas. The strain considering W−Se distances have either compressive or tensile character depending on which side of the metal atoms it resides. The maximum and minimum of the strain on each layer can be read from Table S1. As discussed in the main text, the interlayer distance and stackings also change which influences the band gaps of the system, therefore, the non-monotonic variation of the values is not surprising. A summary of the structural parameters of the homobilayer WSe_2 wrinkle is also presented in Table S2.

Figure S3 shows all the band structure of the systems in this study. A momentum indirect-to-direct transition is observed at approximately 10% of compression. This transition has been already known from the uni- and biaxial strain modulation of TMDCs.[3] However, the out-of-plane variation of strain induces an internal electric field and hence a Rashba-like[4] splitting around Γ (look at Figure S3j). Moreover, as discussed in the main text, the band edges are localized on different layers which correspond to an interlayer exciton IX^{KK}. In order to better quantify the localization of the states, we projected the band structures on different areas of the wrinkle. Table

S3 presents the contribution of the sections to overall band structure. For example the contribution of the upper layer up curve to the CBM+1 is 90.6%. This is related to the figures of the projected band in the main text in which black is equal to 100% and the shades of gray are representative of the values in the table.

The values of different localized band gaps are quantified in Table S4. The apparent band gaps at K is due to the band back folding of the band structure. To better understand the band folding please refer to Supplementary Material (SM) of our previous publication.[5]

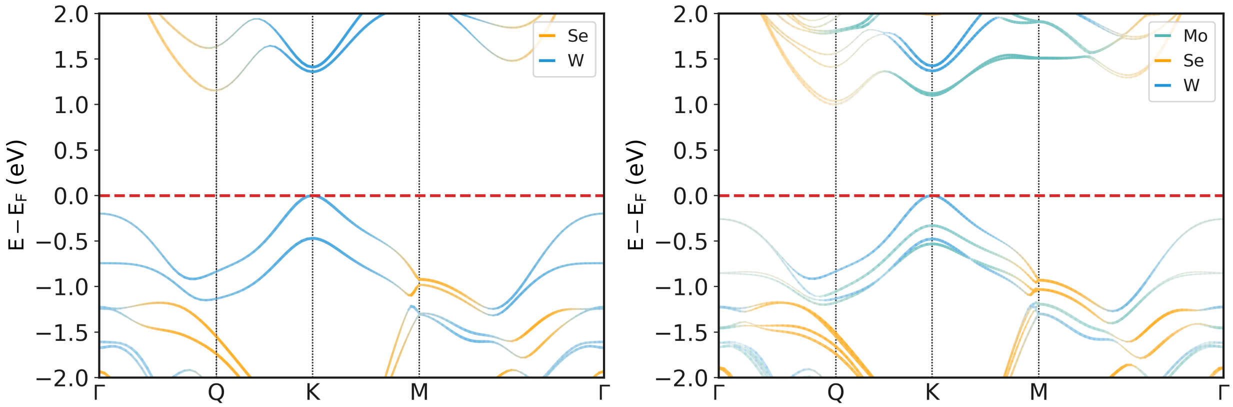

Figure S1: The Mulliken projected band structure of flat, unstrained homobilayer WSe_2 and $WSe_2/MoSe_2$ heterobilayer in hexagonal unit cell. Both structures have an indirect band gap with heterobilayer having smaller difference between direct and indirect band gaps ($E_{\mathrm{indirect}} - E_{\mathrm{direct}}$=76 meV of heterobilayer in comparison to $E_{\mathrm{indirect}} - E_{\mathrm{direct}}$=174 meV of the homobilayer)

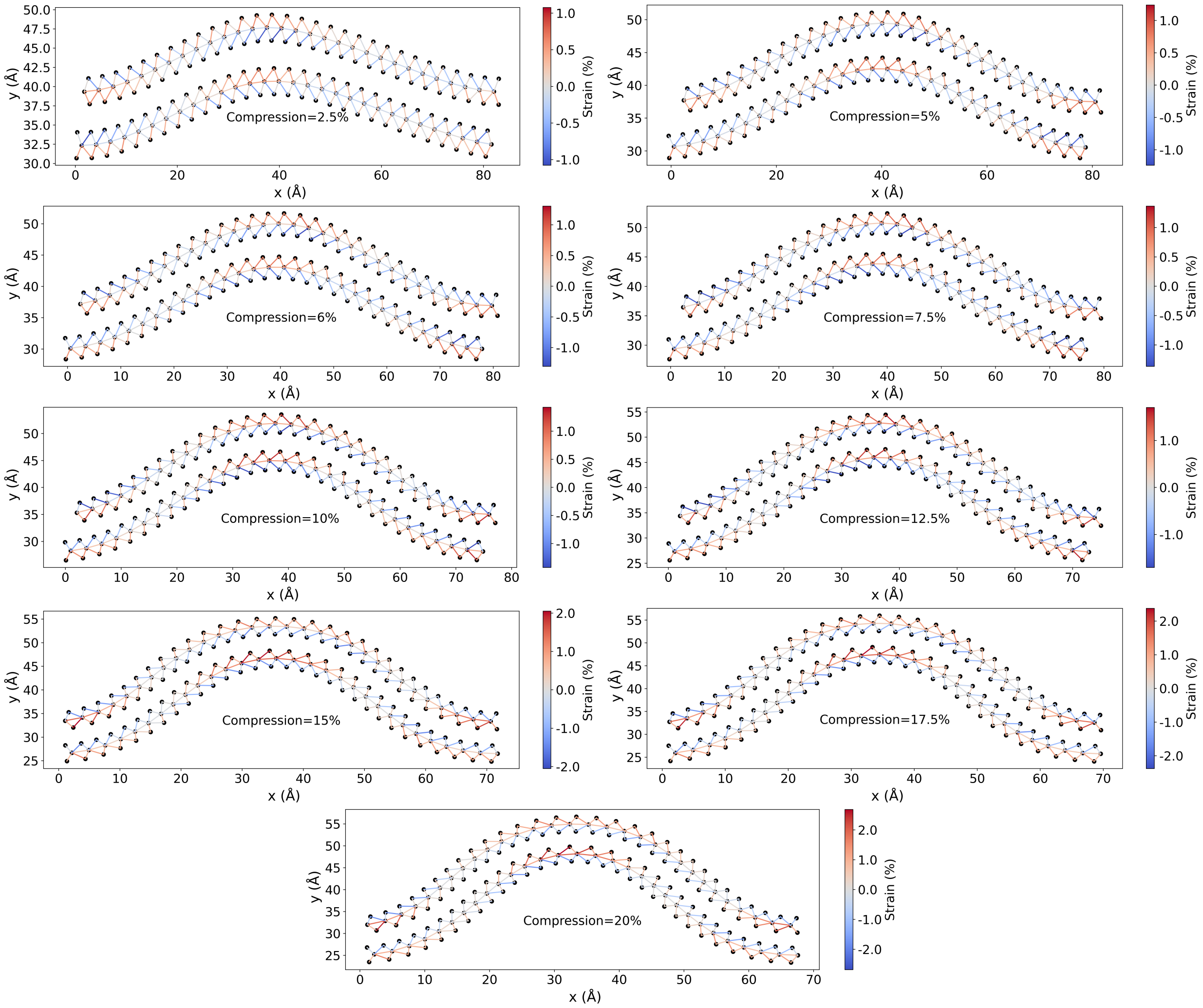

Figure S2: Strain variation along the homobilayer WSe_2 wrinkles. Bond strain for W−W and W−Se bonds, $\varepsilon = (\frac{d-d_{\text{flat}}}{d_{\text{flat}}})\times 100$ with d and d_{flat} are the distance between atoms in the strained and flat, unstrained system

Table S1: Maximum and minimum strain at each layer of the wrinkled homobilayer WSe_2 structure, defined as $\varepsilon = \frac{d-d_{\text{flat}}}{d_{\text{flat}}}$, where d and d_{flat} are the W−W distance in the strained and flat, unstrained system, respectively.

Compression	Maximum strain upper layer	Minimum strain upper layer	Maximum strain lower layer	Minimum strain lower layer
2.5	0.0034	-0.0032	0.0033	-0.0032
5	0.0048	-0.0022	0.0050	-0.0022
6	0.0037	-0.0037	0.0035	-0.0038
7.5	0.0034	-0.0049	0.0033	-0.0049
10	0.0058	-0.0044	0.0059	-0.0044
12.5	0.0077	-0.0063	0.0082	-0.0062
15	0.0135	-0.0018	0.0131	-0.0018
17.5	0.0157	-0.0018	0.0162	-0.0017
20	0.0187	-0.0023	0.0187	-0.0023

Table S2: Structural parameters of the homobilayer WSe_2 in Åfor different compressions- A and radius of curvature, R, values are extracted using the fitted curve explained in the SI

**Compression**	λ	$A_{\text{upper layer}}$	$A_{\text{lower layer}}$	A_{both}	$R_{\text{min}_{\text{upper layer}}}$	$R_{\text{min}_{\text{lower layer}}}$
2.5	83.137	4.171	4.177	7.669	43.54	32.949
5	81.005	5.994	5.982	9.462	28.345	32.929
6	80.152	6.538	6.524	9.997	25.846	32.614
7.5	78.874	7.297	7.280	10.746	22.673	20.623
10	76.741	8.435	8.415	11.875	18.752	16.265
12.5	74.610	9.403	9.383	12.812	16.517	19.125
15	72.479	10.075	10.091	13.494	14.53	11.307
17.5	70.347	10.857	10.872	14.275	12.863	9.662
20	68.215	11.562	11.582	14.973	8.637	8.444

Table S3: Contribution of the atoms of different sections of the 20% compressed WSe_2 homobilayer wrinkle to different bands at the reciprocal lattice K. "100" would indicate that the band is only composed of states from this section. Please refer to the Figure 3 for the section's location.

**Band index**	**Upper layer straight**	**Lower layer straight**	**Upper layer up curve**	**Upper layer down curve**	**Lower layer up curve**	**Lower layer down curve**
**CBM+1**	8.7%	0.1%	0.2%	90.6%	0.0 %	0.3%
**CBM**	0.1%	8%	0.3%	0. %	91.3%	0.3%
**VBM**	0.1%	15%	0.0%	0.3%	1.5%	83.1%
**VBM-1**	18.2%	0.1%	79.9%	1.5%	0.3%	0.%

Table S4: Different local band gaps in eV of homobolayer WSe_2 wrinkles at the reciprocal space K point for different compression. a) Interlayer band gap IX^{KK}, b) the smallest apparent (interlayer) band gap at K. Intralayer bands gaps: c) upper layer down curve, d) upper layer straight, e) upper layer up curve, f) lower layer down curve, g) lower layer straight, h) lower layer up curve (at least 30% localization in the respective region).

**Compression**	**a**	**b**	**c**	**d**	**e**	**f**	**g**	**h**
2.5	1.272	1.204	2.386	1.252	1.35	1.35	1.204	1.794
5	1.245	1.196	1.325	1.196	1.342	1.342	1.196	1.325
6	1.198	1.198	1.336	1.198	1.397	1.397	1.198	1.277
7.5	1.197	1.197	1.279	1.197	1.334	1.334	1.198	1.279
10	1.193	1.193	1.316	1.226	1.318	1.318	1.226	1.283
12.5	1.192	1.192	1.287	1.289	1.306	1.305	1.289	1.287
15	1.128	1.128	1.246	1.281	1.307	1.307	1.281	1.246
17.5	1.11	1.11	1.233	1.245	1.295	1.294	1.245	1.232
20	1.094	1.094	1.22	1.28	1.281	1.281	1.28	1.219

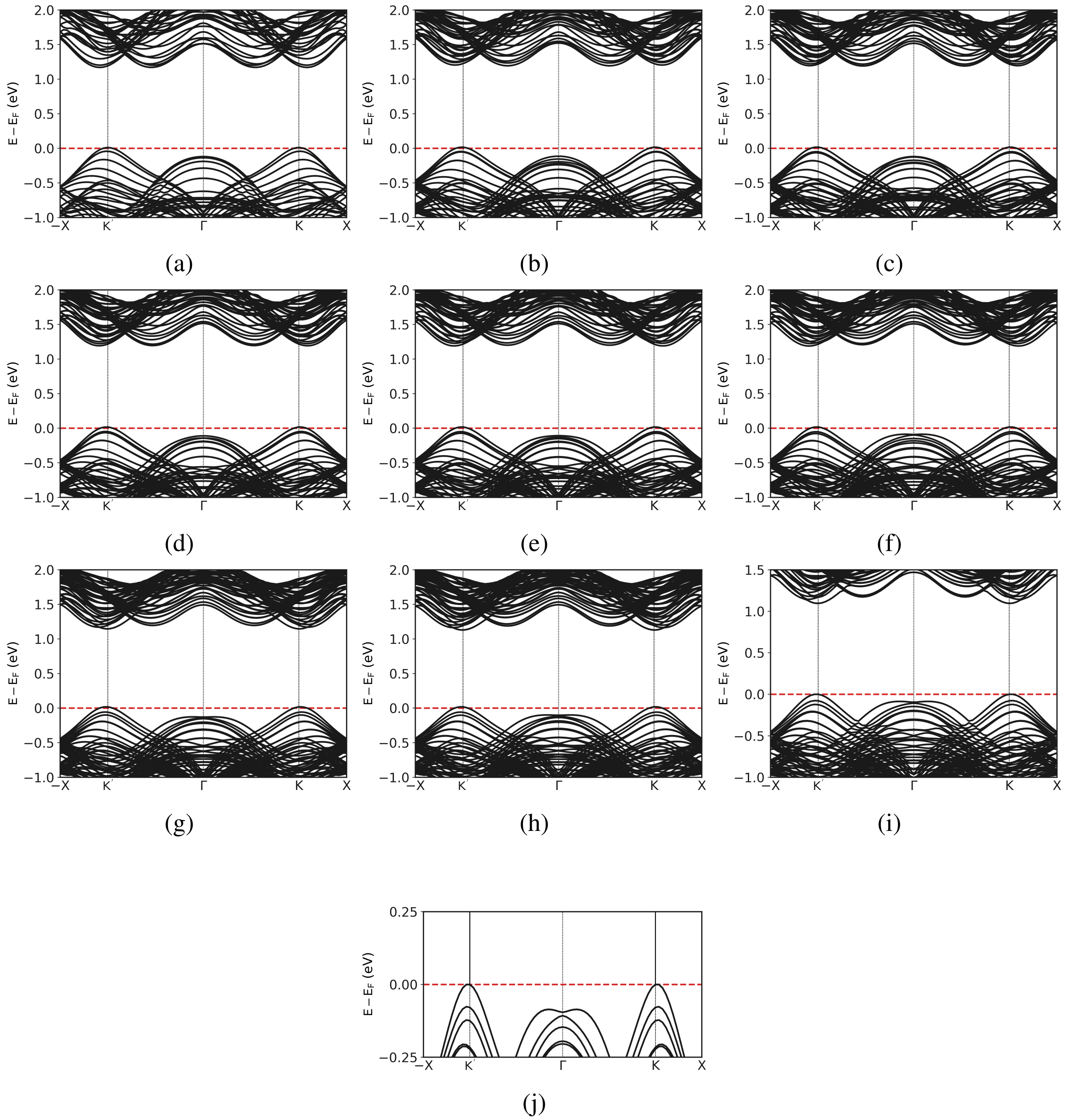

Figure S3: Band structure changes in homobilayer WSe_2 due to wrinkling at different compressions of a) 0% b) 2.5% c) 5% d) 7.5% e) 10% f)12.5% g) 15% h)17.5% i) 20% j)20% enlarged area of the band structure of 20% compressed system. Rashba-like splitting is well apparent. Band gaps of the systems are reduces with strain with the CB at K being more sensitive to strain. An indirect to direct transition occurs also for the higher strained systems.

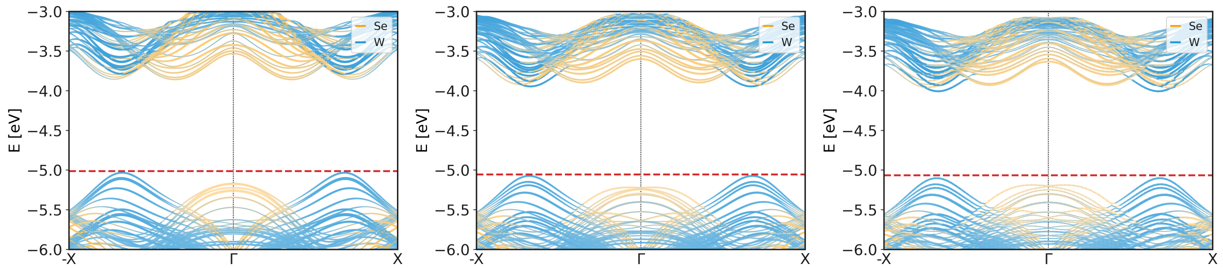

Figure S4: Band structure of WSe_2 homobilayer for different compressions (5%, 10% and 20%) without shifting of the energy values. Bands shift downwards, however the shift of the conduction band is larger thus leading to a reduction of the band gap.

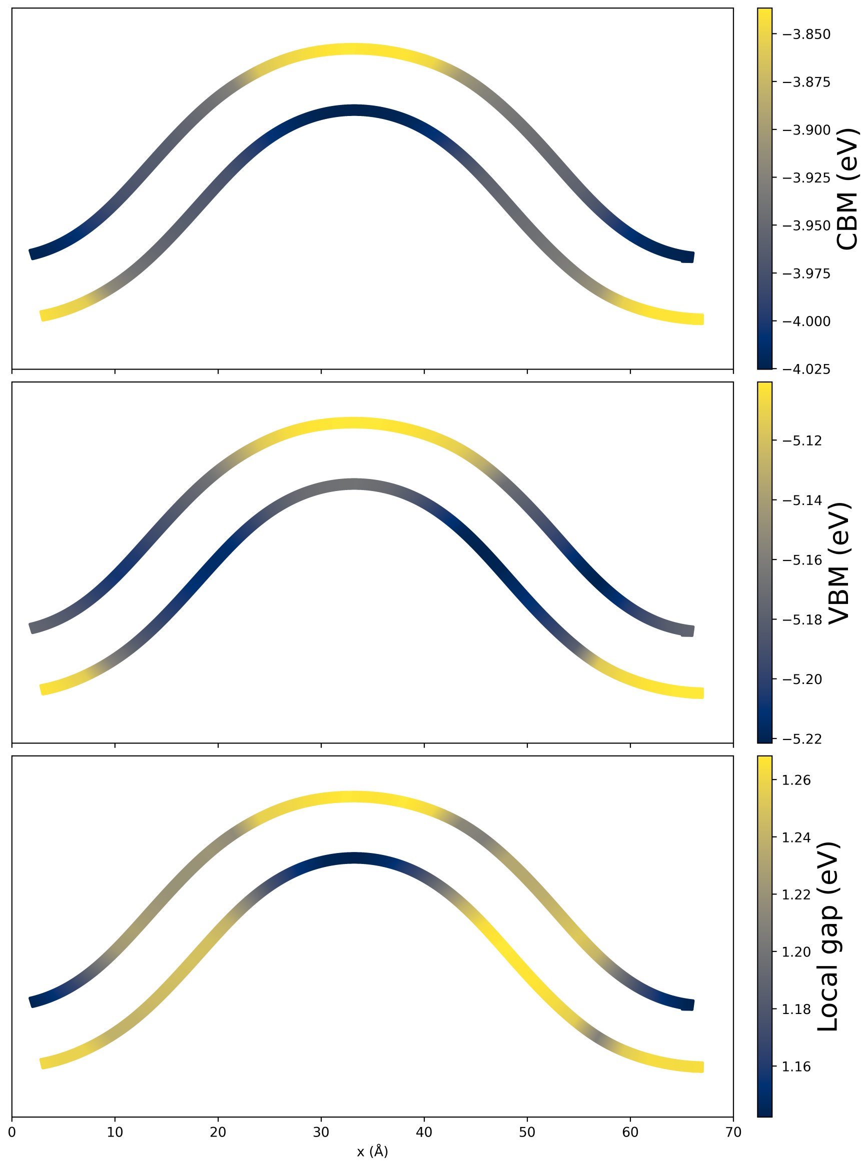

Figure S5: Distribution of band gap and electronic structure edges (CBM and VBM) of 20% compressed wrinkled homobilayer of WSe_2.

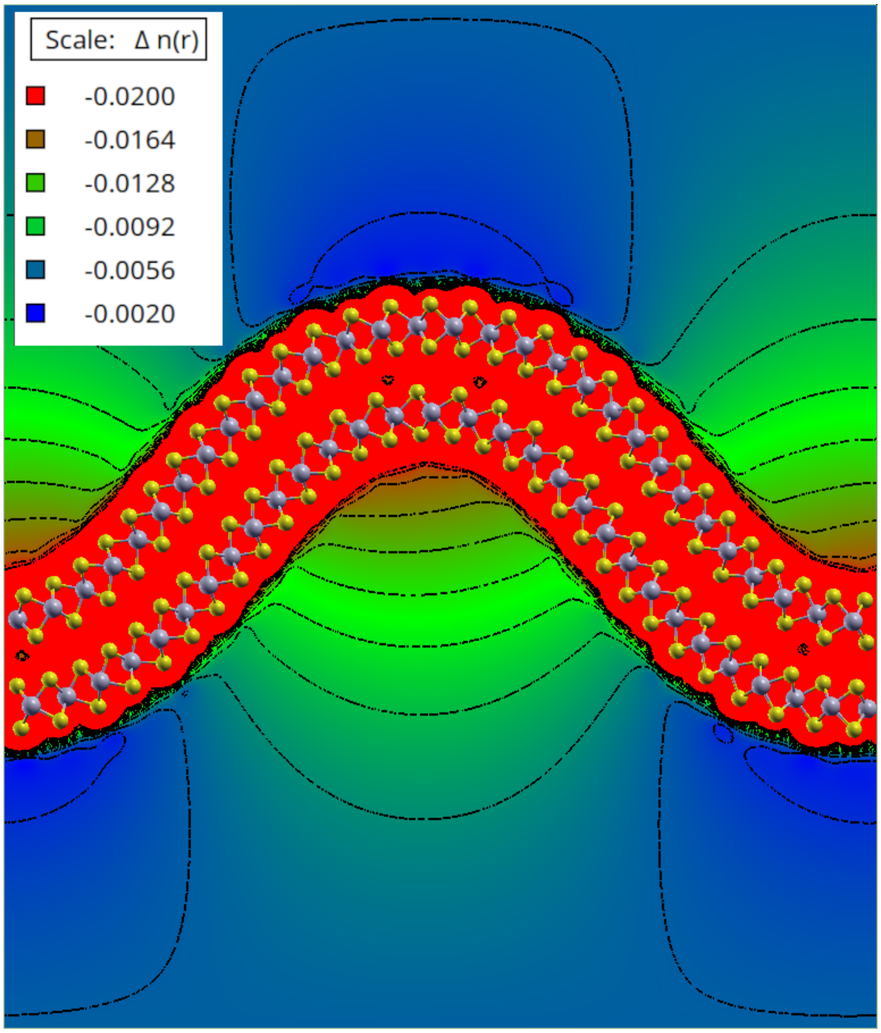

Figure S6: Hartree potential variation in unit of Ha of 20% compressed wrinkled homobilayer of WSe_2.

Momentum-matrix elements

In order to estimate the changes in the oscillator strength of the intra- and interlayer excitons, we calculated the momentum-matrix elements[6,7] (MMEs) for the flat and 20%-compressed homo- and heterobilayers. The following tables summarize the MMEs for the most important band-edge transitions and also classify them as intra-/interlayer transitions and whether they are spin-conserving (the expectation value $\langle\sigma_z\rangle$ has the same sign for initial and final state) or spin-flip transitions. We furthermore show the MMEs for some transitions along high-symmetry lines for the case of flat and wrinkled homobilayers in Figs. S7–S10 and for the wrinkled heterobilayer in Fig. S11. Most notably, the MME for the spin-conserving, intralayer transition using in-plane circularly polarized light reduces by one order of magnitude when going from the flat (VB→CB+3, 2.66e-1 $\hbar/a_0$) to the wrinkled homobilayer (VB→CB+2, 6.31e-2 $\hbar/a_0$). This is due to both states being localized in different curves of the wrinkle. Furthermore, the MMEs for out-of-plane polarized light increase by orders of magnitude for the spin-conserving intralayer and the spin-flip interlayer transitions – e.g., from 2.60e-7 $\hbar/a_0$ for VB→CB+2 (flat, spin-flip, inter) to 2.12e-3 $\hbar/a_0$ for VB→CB+3 (wrinkled). This is in stark contrast to in-plane circularly polarized light for which the MME for the spin-flip interlayer transition is slightly larger than the spin-conserving interlayer transitions. Both have the same order of magnitude as the spin-flip intralayer transition which might be due to the fact that both transitions are energetically degenerate with intralayer transition.

The analysis for the wrinkled $WSe_2/MoSe_2$ heterobilayer is not as easy, especially for the intralayer transition, since the VBM of $MoSe_2$ and the CBM of WSe_2 are hidden by states of the other layer due to the type-II band alignment. In Fig. S11 we show the MMEs for the four transitions which we think can be related to the spin-flip and -conserving intra- and interlayer transition.

Table S5: Calculated MMEs (in units of $\hbar/a_0$) at the K point for in-plane, circularly polarized (first two columns, left-handed + right-handed polarization, "$\circlearrowleft + \circlearrowright$") and out-of-plane polarized light (last two columns, "$\perp$") for the flat WSe_2 homobilayer.

	$\circlearrowleft$+$\circlearrowright$		$\perp$	
**Final\Initial state**	VB-1	VB	VB-1	VB
CB	2.58e-2	1.14e-2	6.20e-3	1.46e-2
CB+1	1.12e-2	2.59e-2	1.46e-2	6.20e-3
CB+2	2.66e-1	9.10e-2	3.08e-6	2.60e-7
CB+3	9.10e-2	2.66e-1	8.55e-7	5.56e-6

Table S6: Classification of the transitions for the flat WSe_2 homobilayer.

**Final\Initial state**	VB-1	VB
CB	spin-conserving, inter	spin-flip, intra
CB+1	spin-flip, intra	spin-conserving, inter
CB+2	spin-conserving, intra	spin-flip, inter
CB+3	spin-flip, inter	spin-conserving, intra

Table S7: Calculated MMEs (in units of $\hbar/a_0$) at the K point for in-plane, circularly polarized (first two columns, left-handed + right-handed polarization, "$\circlearrowleft + \circlearrowright$") and out-of-plane polarized light (last two columns, "$\perp$") for the 20%-compressed WSe_2 homobilayer.

	$\circlearrowleft$+$\circlearrowright$		$\perp$	
**Final\Initial state**	VB-1	VB	VB-1	VB
CB	4.87e-3	2.05e-2	3.48e-3	1.22e-2
CB+1	2.06e-2	4.87e-3	1.22e-2	3.53e-3
CB+2	6.20e-3	6.31e-2	2.23e-3	8.60e-3
CB+3	6.38e-2	6.24e-3	9.00e-3	2.12e-3

Table S8: Classification of the transitions for the 20%-compressed WSe_2 homobilayer.

**Final\Initial state**	VB-1	VB
CB	spin-conserving, inter	spin-flip, intra
CB+1	spin-flip, intra	spin-conserving, inter
CB+2	spin-flip, inter	spin-conserving, intra
CB+3	spin-conserving, intra	spin-flip, inter

Table S9: Calculated MMEs (in units of $\hbar/a_0$) at the K point for in-plane, circularly polarized (first four columns, left-handed + right-handed polarization, "$\circlearrowleft + \circlearrowright$") and out-of-plane polarized light (last four columns, "$\perp$") for the flat $WSe_2/MoSe_2$ heterobilayer.

	$\circlearrowleft$+$\circlearrowright$				$\perp$			
**Final\Initial state**	VB-3	VB-2	VB-1	VB	VB-3	VB-2	VB-1	VB
CB	9.87e-4	7.11e-2	2.14e-1	6.98e-3	4.37e-6	3.47e-7	5.87e-7	1.28e-6
CB+1	2.24e-1	6.03e-6	8.90e-6	2.28e-2	3.25e-7	6.15e-3	8.34e-3	4.62e-7
CB+2	3.49e-6	2.57e-1	8.62e-2	4.20e-6	1.46e-2	3.65e-7	7.11e-7	2.48e-2
CB+3	2.66e-2	3.21e-3	7.36e-3	2.83e-1	1.67e-7	2.25e-6	2.34e-6	4.33e-7

Table S10: Classification of the transitions for the flat $WSe_2/MoSe_2$ heterobilayer as spin-flip (sf) and spin-conserving (sc) intra- and interlayer transitions.

**Final\Initial state**	VB-3	VB-2	VB-1	VB
CB	sf, intra	sc, inter	sc, intra	sf, inter
CB+1	sc, intra	sf, inter	sf, intra	sc, inter
CB+2	sf, inter	sc, intra	sc, inter	sf, intra
CB+3	sc, inter	sf, intra	sf, inter	sc, intra

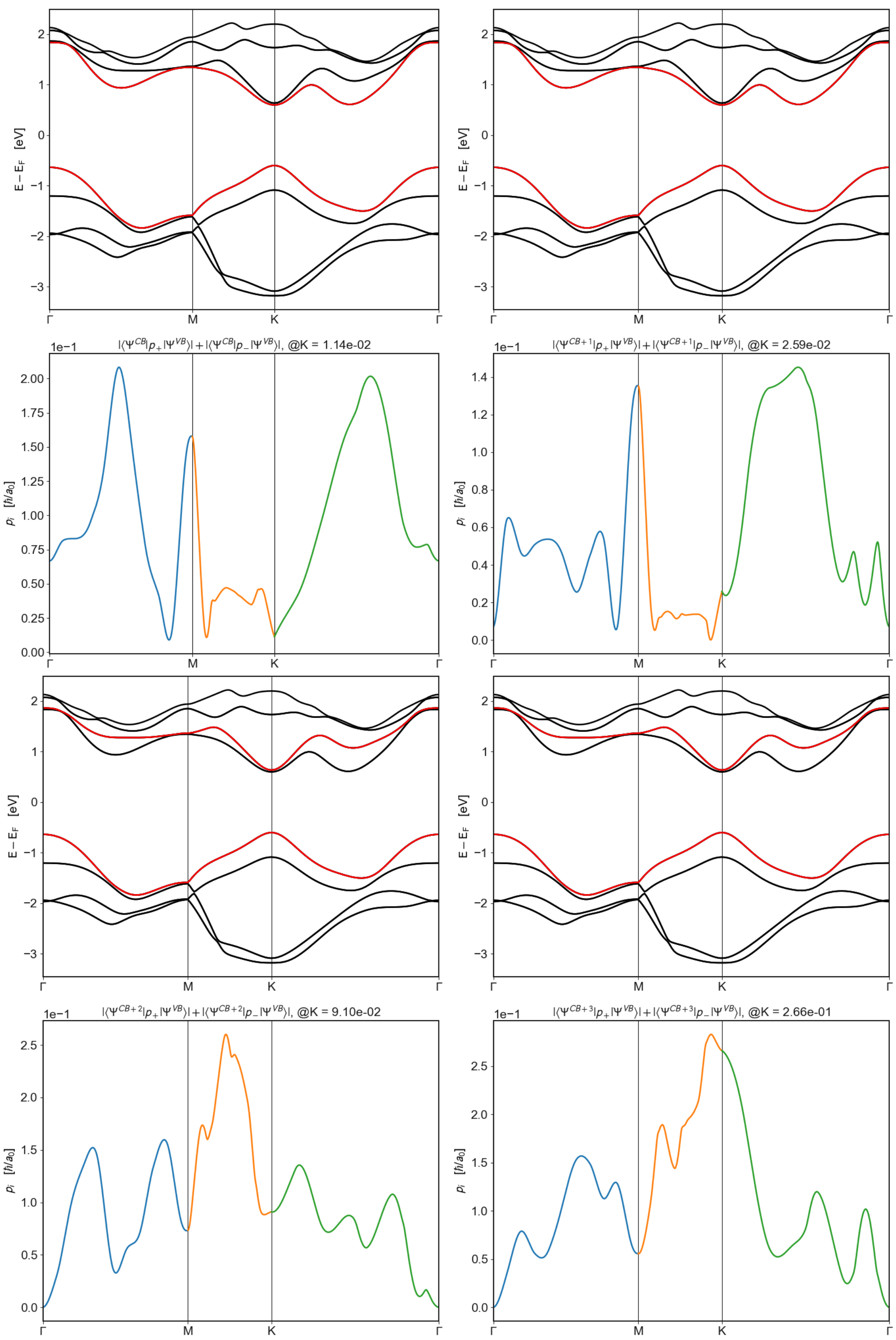

Figure S7: Sum of the calculated MMEs for left- and right-handed, in-plane polarized light along Γ→M→K→Γ for the flat WSe_2 homobilayer. Shown are the four most important transitions with the valence band as initial state. The initial and final state are highlighted in red in the band structure.

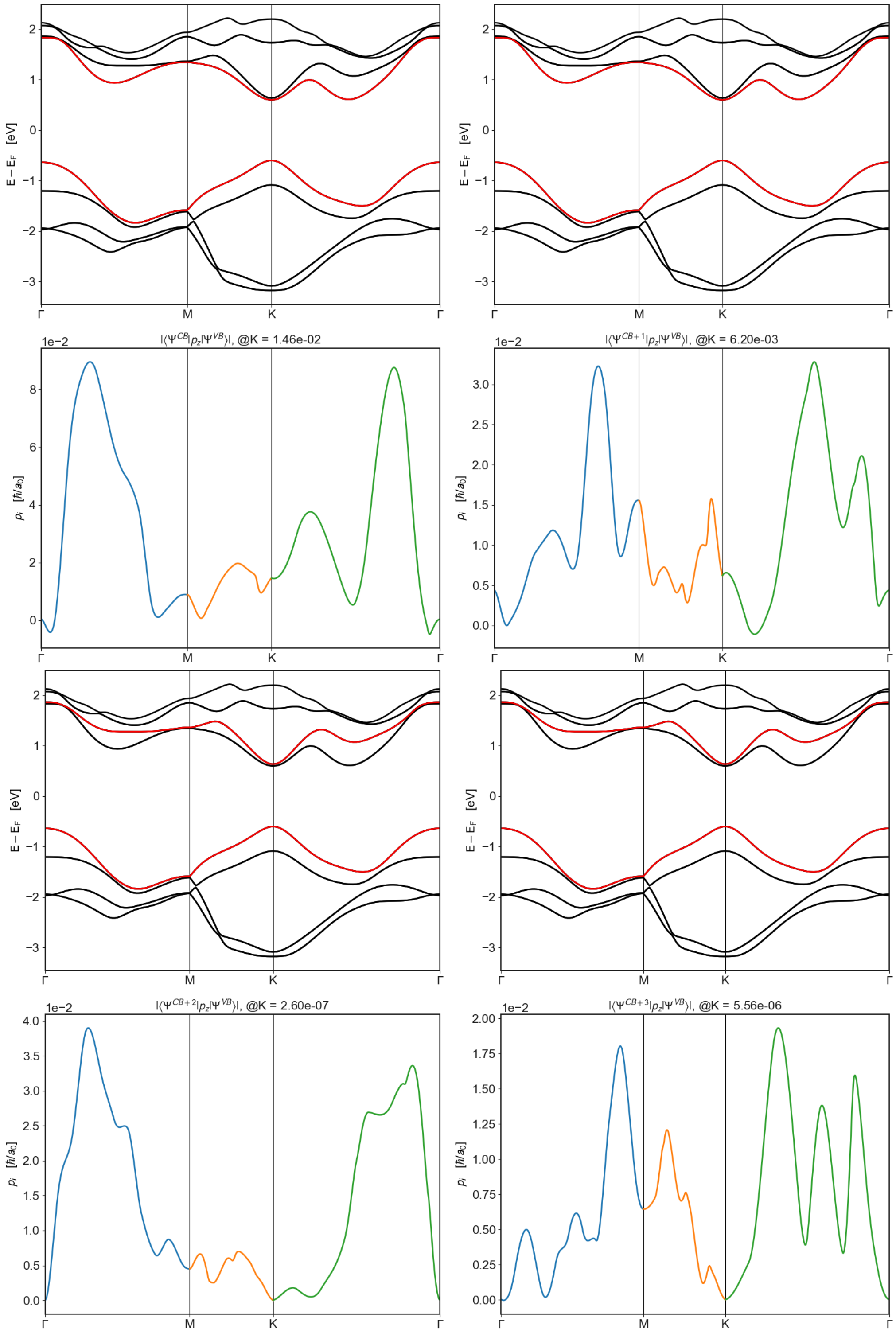

Figure S8: Same as Fig. S7 but for out-of-plane polarized light.

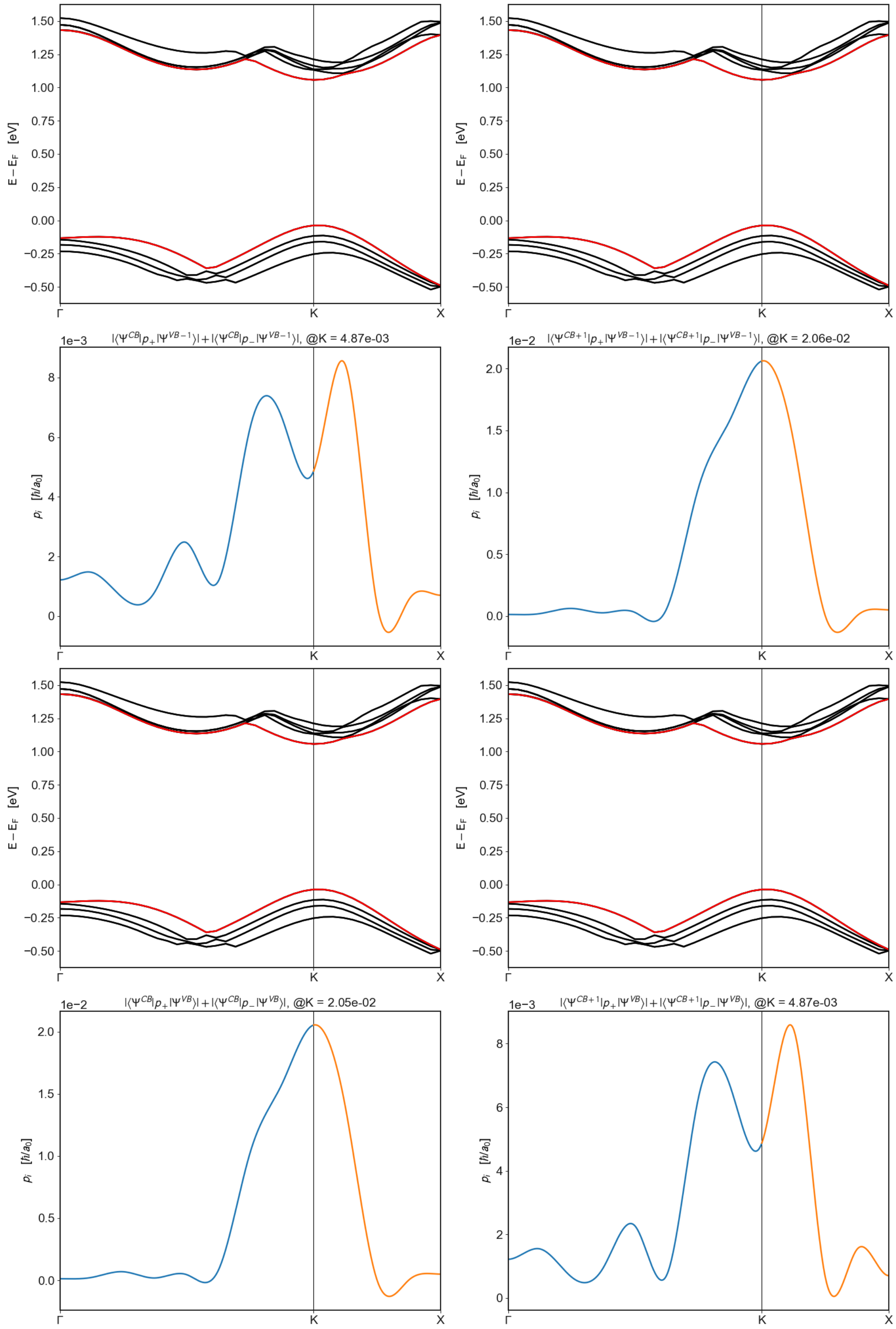

Figure S9: Same as Fig. S7 but for the wrinkled WSe_2 homobilayer and along the Γ→X line.

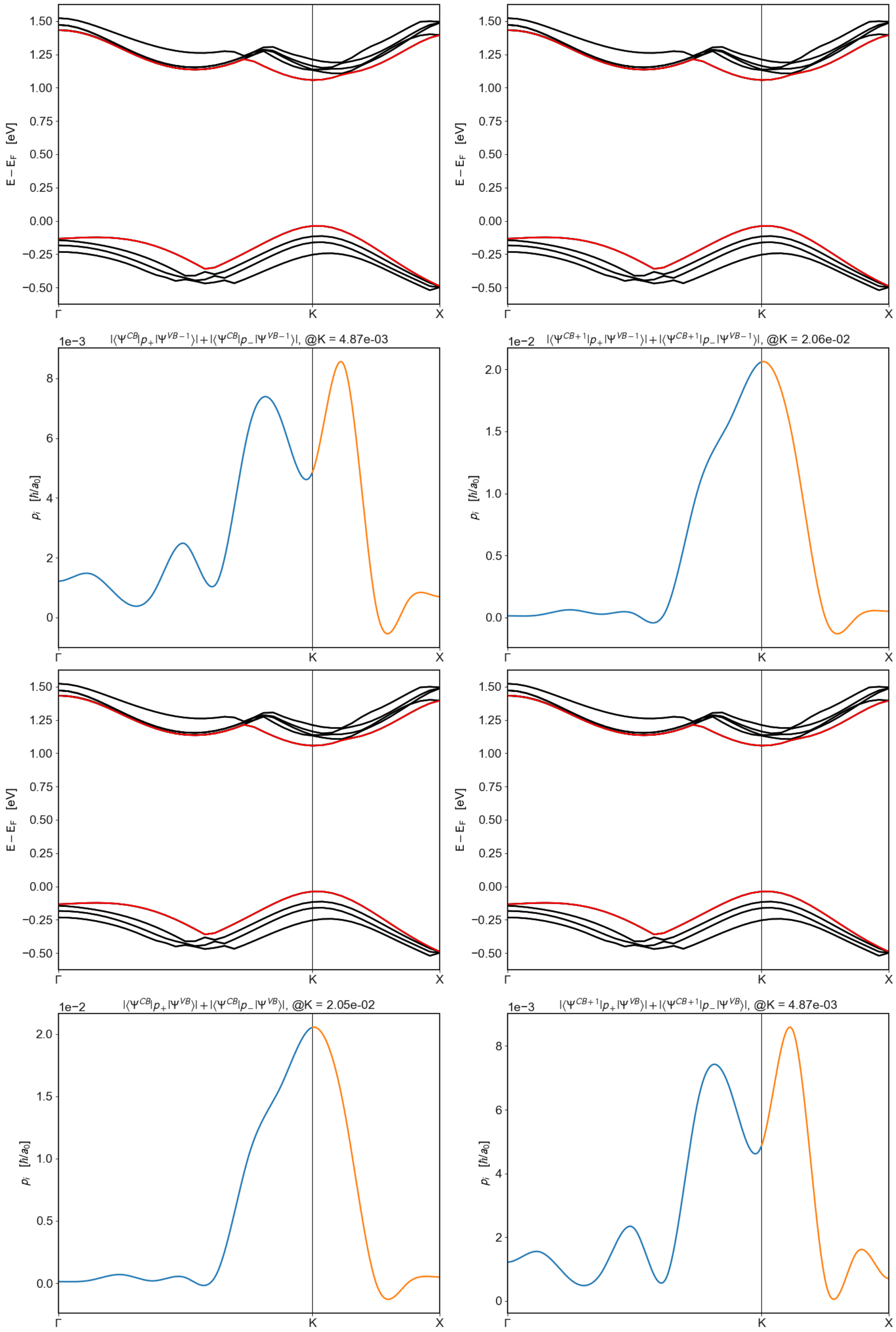

Figure S10: Same as Fig. S9 but for out-of-plane polarized light.

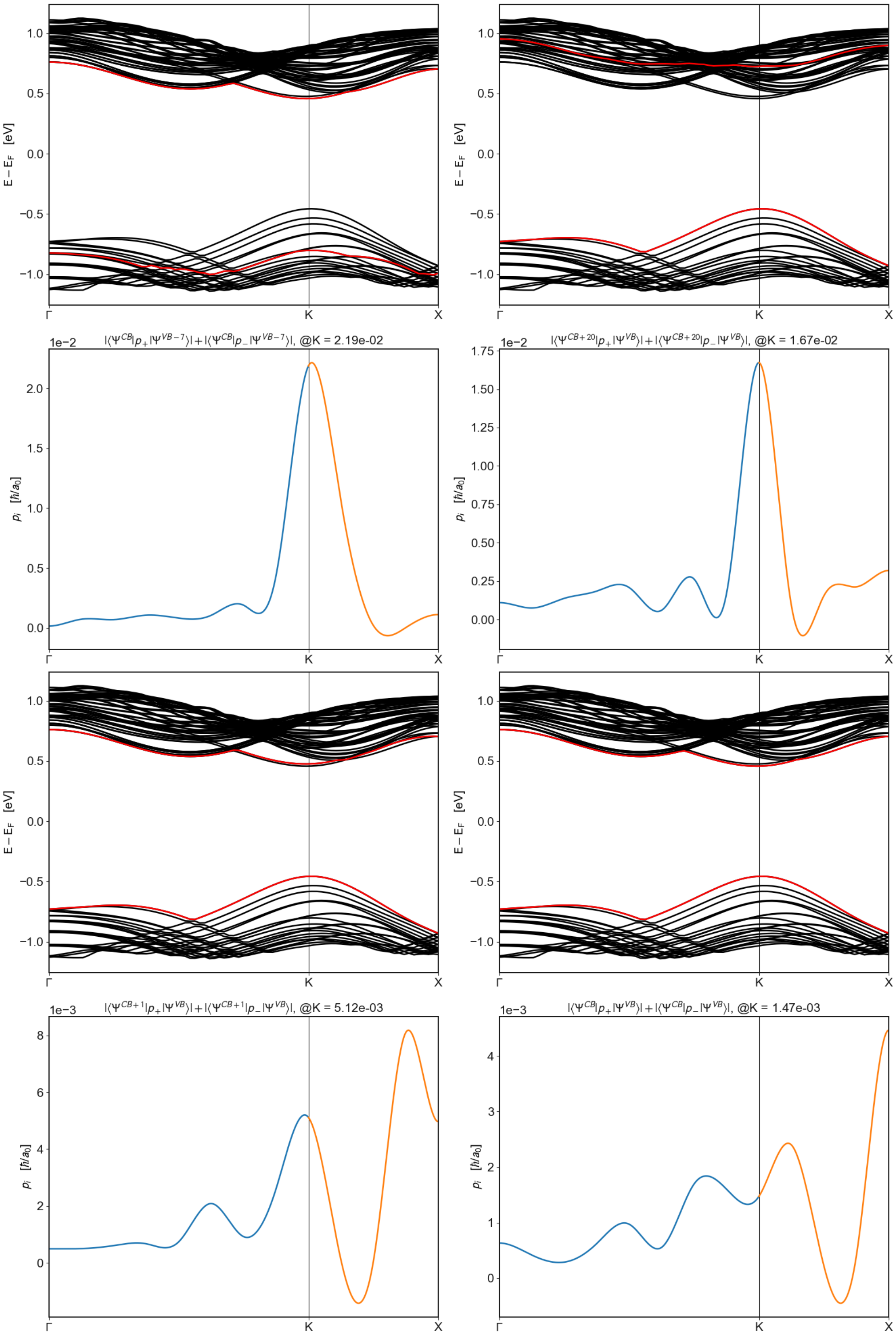

Figure S11: Same as Fig. S9 but for the wrinkled $WSe_2/MoSe_2$ heterobilayer. Shown are the spin-conserving intralayer transitions for $MoSe_2$ (upper left) and WSe_2 (upper right), as well as the spin-conserving and spin-flip interlayer transitions (lower left and right).

Stacking effect in homobilayer WSe_2

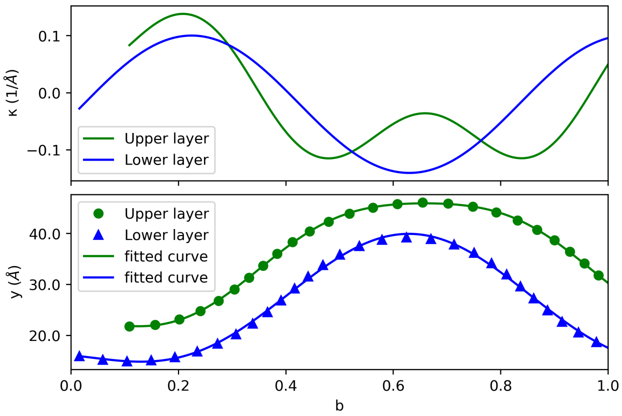

Figure S12: Structural information of the R_h^h stacking homobilayer WSe_2 under compression. Curvature, κ , and the atomic positions of the W atoms of wrinkled R_h^h WSe_2 (lower panel) for 20% compression. b is the unit vector of the lattice parameter in the direction of the wrinkle

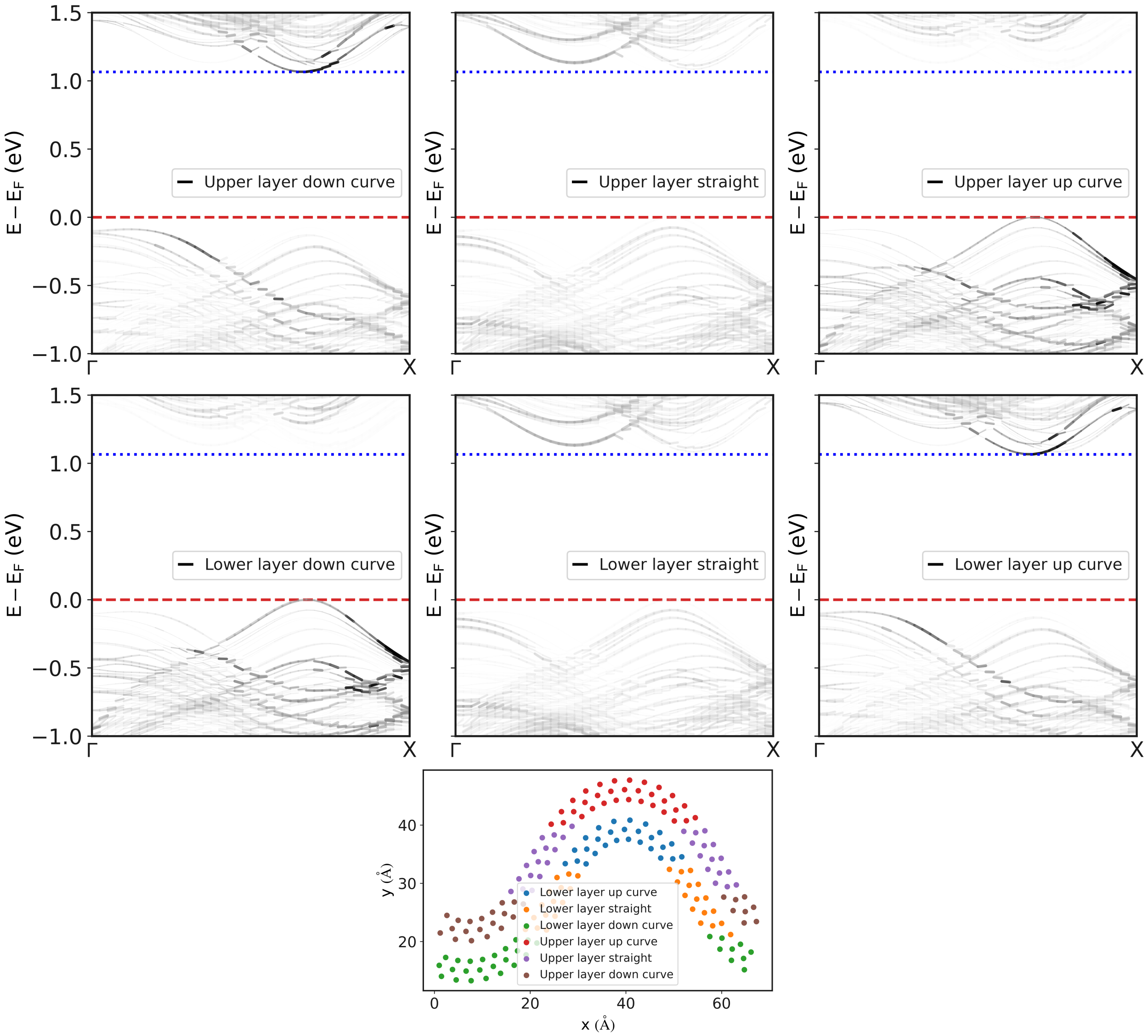

Figure S13: Contribution of different sections of the wrinkled WSe_2 with a R_h^h stacking at 20% compression, position of contributing atoms are depicted below

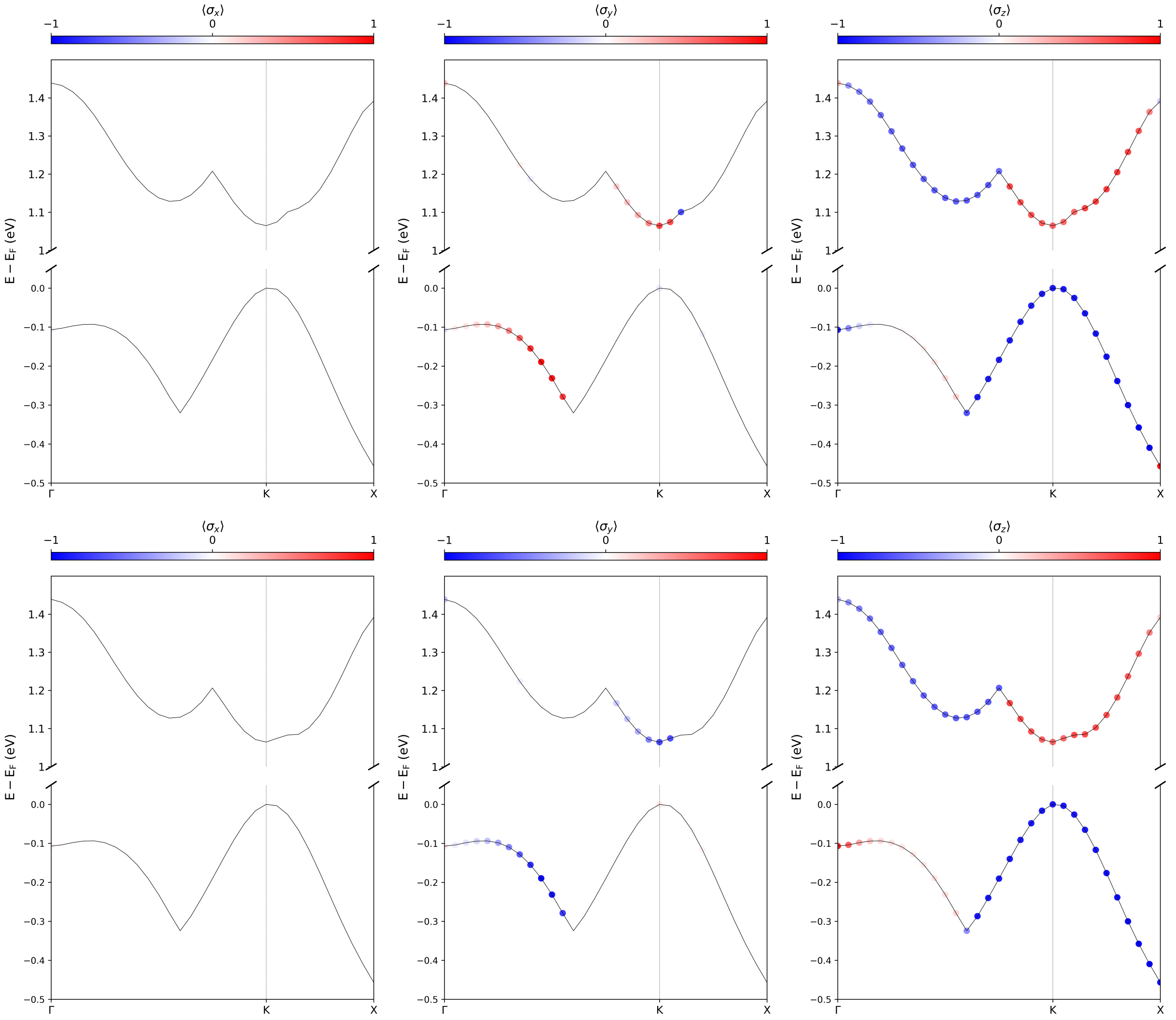

Figure S14: The valence and conduction bands of wrinkled R_h^h homobilayer WSe_2 at 20% compression. The first row depicts the expectation values $\langle\sigma_i\rangle$, i.e. the spin texture, of VB and CB+1 states localized in the same curved region but on different layers. The second row shows their energy degenerate partners (VB-1 and CB) localized on the other curved region.

Size effect of homobilayer WSe_2

We also performed an additional calculation, similar to that described in the Methods section, using a smaller homobilayer to analyze the effect of the initial system size on our conclusions. Specifically, we started from a flat WSe_2 homobilayer comprising a $1 \times 10 \times 1$ rectangular supercell. Upon gradually compressing the system, wrinkles again formed in the structure. However, for the same applied compression, the corresponding strain is higher in this smaller system. Despite this difference, the localization of the band edges follows the same trends as observed in the larger structures.

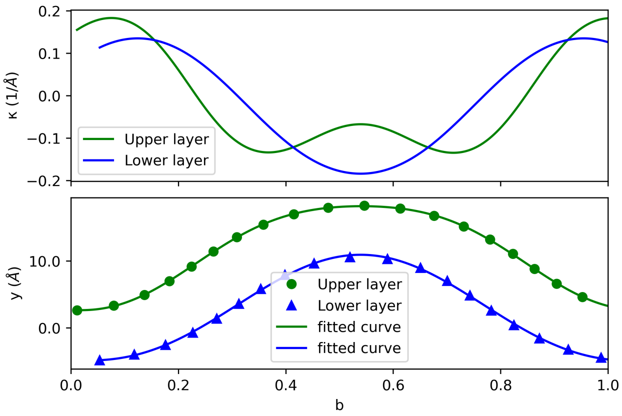

Figure S15: Structural information of the homobilayer H_h^h WSe_2 consisting of 10 unit cells under compression. Curvature, κ, and the atomic positions of the W atoms of wrinkled H_h^h WSe_2 (lower panel) for 20% compression. b is the unit vector of the lattice parameter in the direction of the wrinkle

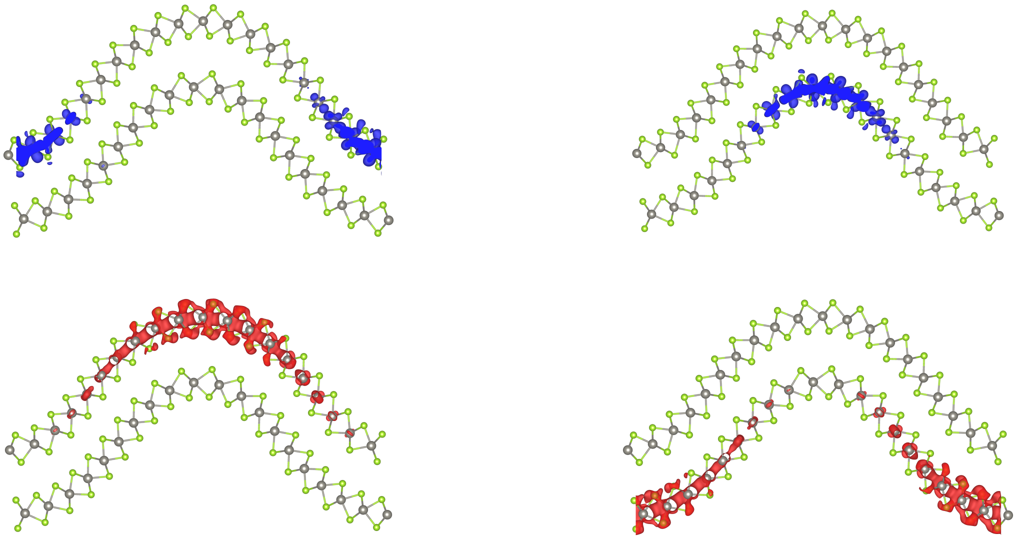

Figure S16: The initial size of flat system does not influence the conclusion of the paper. lectron and holes are localized in different spatial positions of the bilayer wrinkle, giving rise to the formation of interlayer excitons in homobilayer WSe_2. The VB-1, VB, CB and CB+1 eigenstate density $|\psi|^2$ are depicted to show electron localization in blue (up) and hole localization in red (below) for the 20% compressed homobilayer WSe_2.

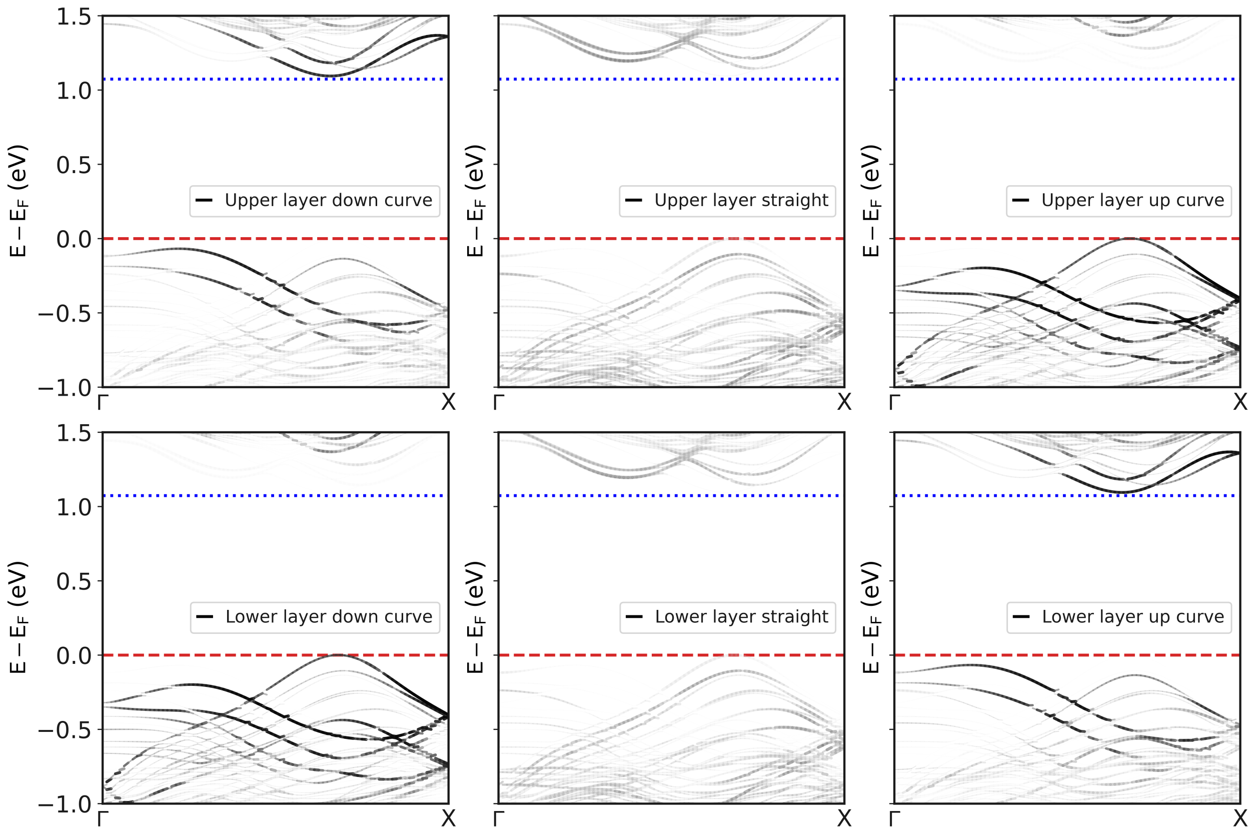

Figure S17: Contribution of different sections of the 10 unit cell wrinkled WSe_2 with a H_h^h stacking at 20% compression, position of contributing atoms are depicted below

Homobilayer $MoSe_2$

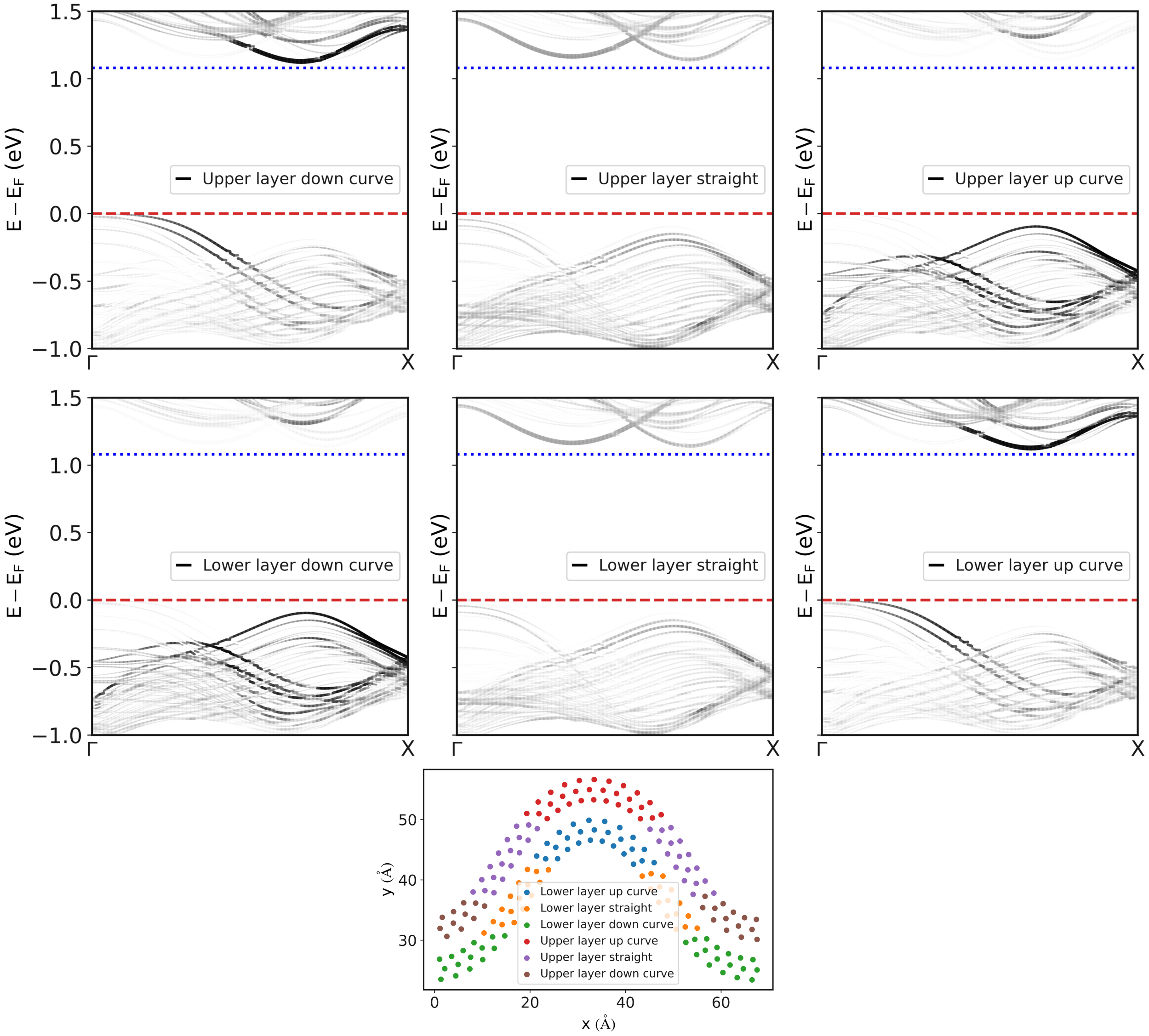

Figure S18: Contribution of different sections of the wrinkled $MoSe_2$ at 20 % compression, position of contributing atoms are depicted below

Spin texture in WSe_2 bilayer wrinkles

Figure S19 shows the spin texture of the for VBM-1, VBM, CBM and CBM+1 for the 20% compression. Strain leads to the localization of the VBMs and CBMs within different regions of the structure – especially the formation of a local, interlayer band gap with both band edges having the same spin expectation value is important. Figures S20 and S21 show the spin texture for all bands close to Fermi level. It should be noted that due to energy degeneracy of the band some of the information is hidden and figure S19 should be consulted.

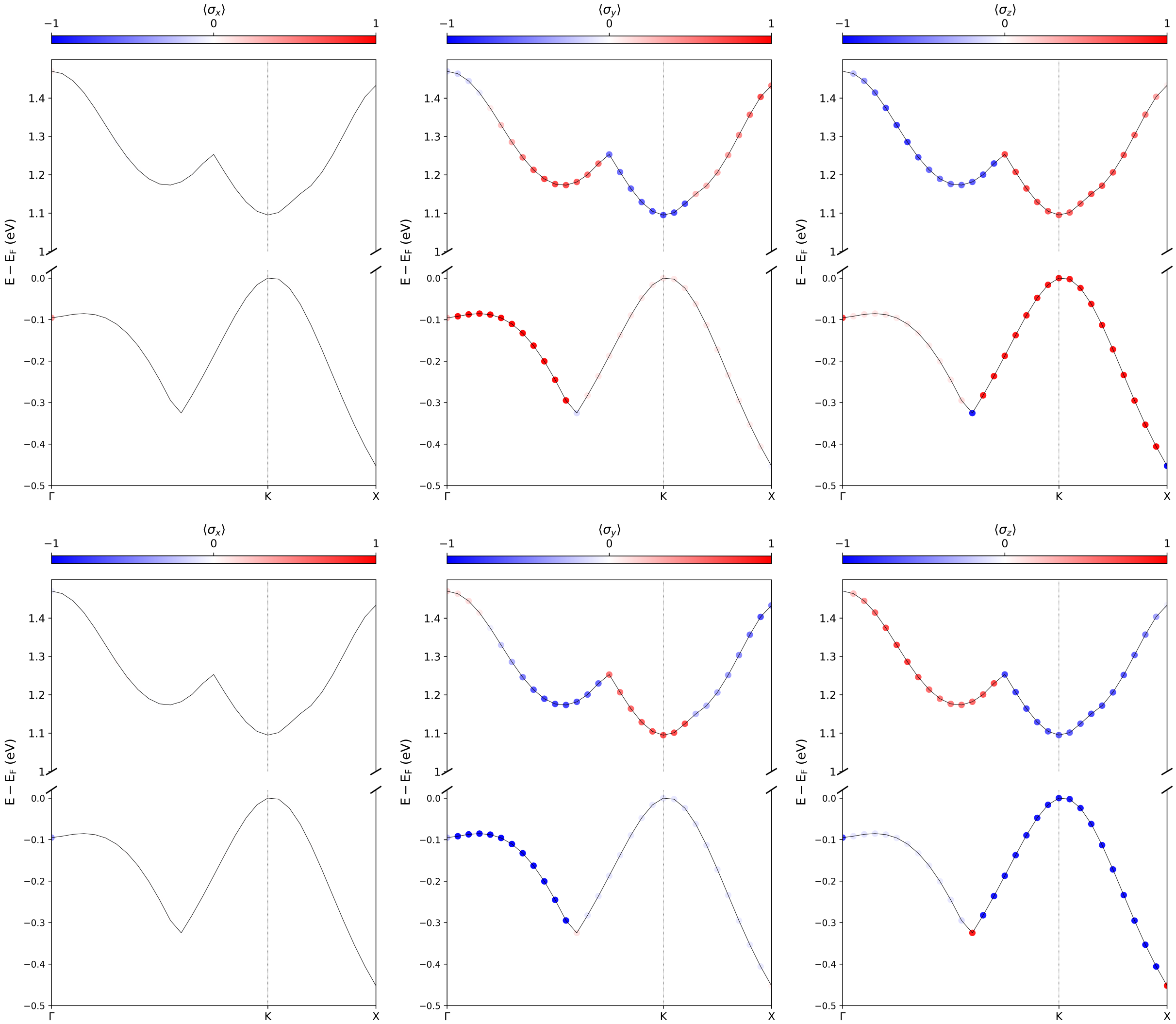

Figure S19: Valence and conduction bands of the homobilayer WSe_2 are made of two energy-degenerate bands with opposite spin directions, each localized on a different layer.[8] The first row depicts the expectation values $\langle \sigma_i \rangle$, i.e. the spin texture, of VB and CB+1 states localized in the same curved region but on different layers. The second row shows their energy degenerate partners (VB-1 and CB) localized on the other curved region. Plots are for the homobilayer wrinkle at 20% compression.

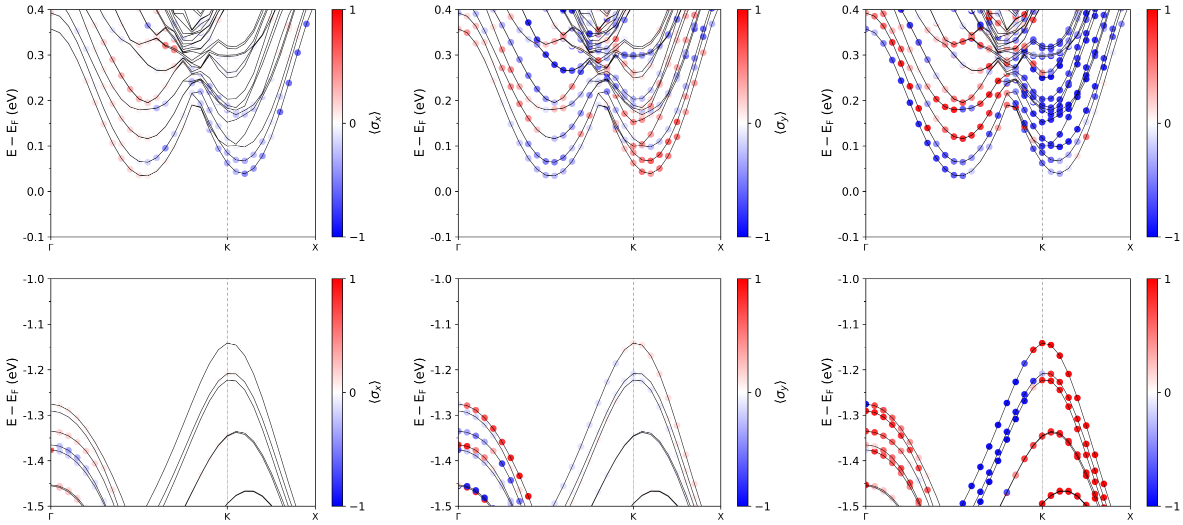

Figure S20: Expectation values of the Pauli matrices $\langle \sigma_i \rangle$ of the wrinkled WSe_2 homobilayer at 2.5% compression.

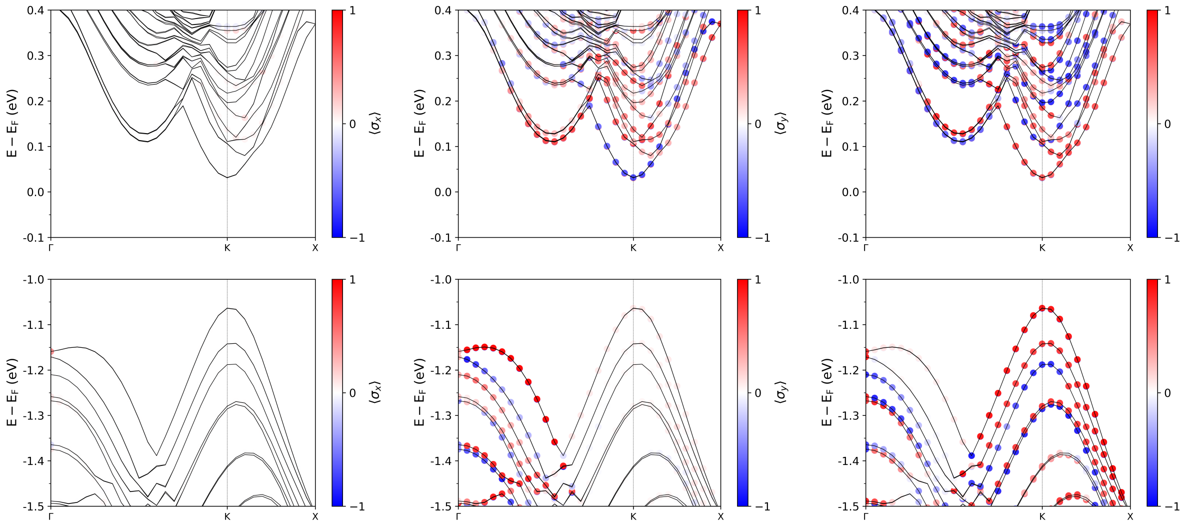

Figure S21: Expectation values of the Pauli matrices $\langle \sigma_i \rangle$ of the wrinkled WSe_2 homobilayer at 20% compression.

$WSe_2/MoSe_2$ heterobilayer wrinkle

In this section, we provide information for the heterobilayer systems similar to homobilayer WSe_2. Figure S22 shows the band structures of the heterostructure systems in this study. The band gap decreases with increasing strain and an indirect-to-direct band gap transition occurs. In heterobilayers, the band edges are localized on different layers and an interlayer exciton has the lowest energy. Wrinkling also releases some of the deformation energy after compression of the 2D surface. Figure S23 shows the system at its local minimum before and after wrinkling. Figure S24 shows the Mulliken bands of the wrinkle under 2.5% compression, projected on different areas of the wrinkle (the figure for the wrinkles under 20% compression can be found in the main text). In contrast to the wrinkle under 20% compression, the electron and hole edges are not localized on different peaks and valleys of the wrinkle.

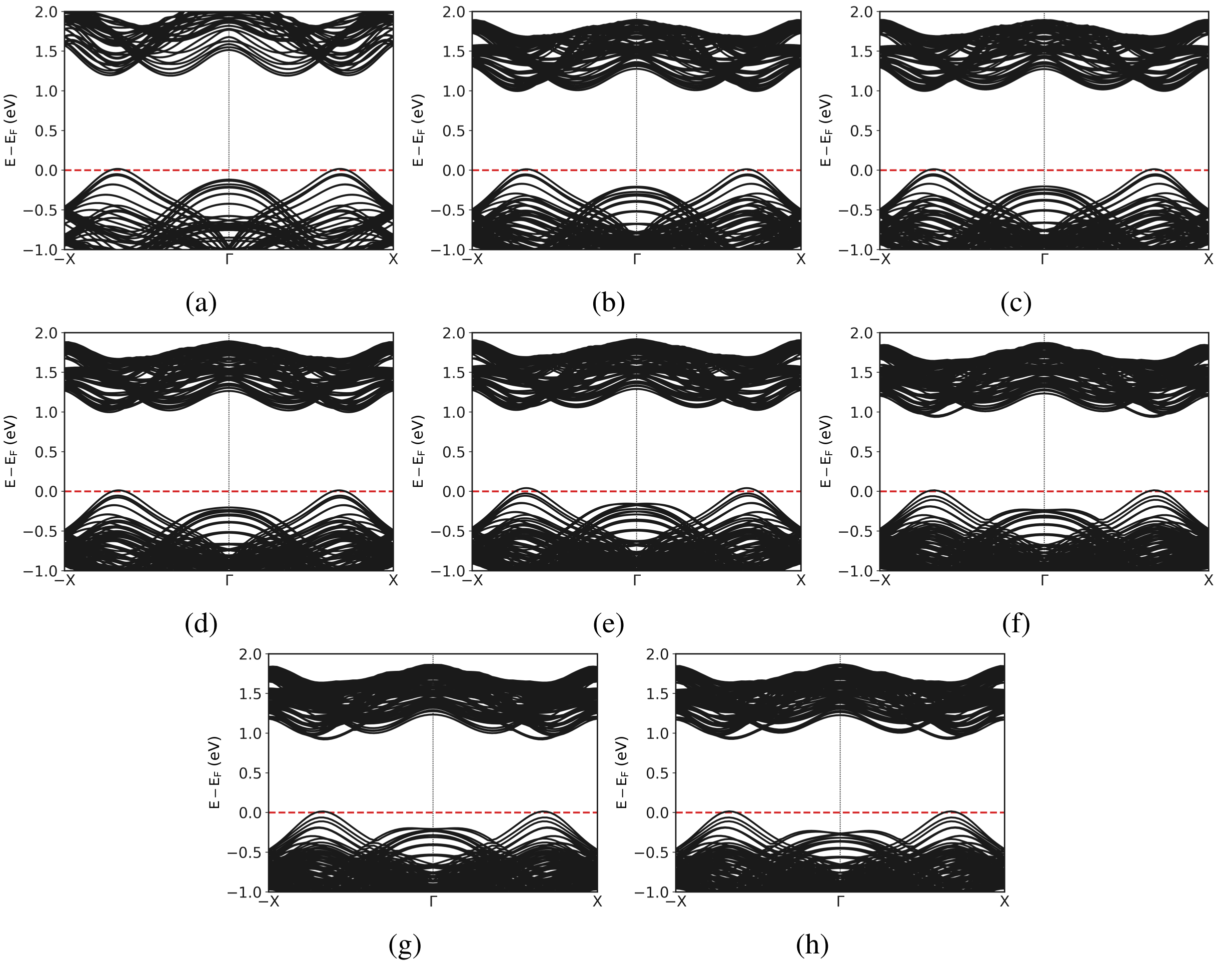

Figure S22: Band structure of $WSe_2/MoSe_2$ heterobilayer for different compressions a) 2.5% b) 5% c) 7.5% d) 10% e) 12.5 f) 15% g)17.5% h) 20%.

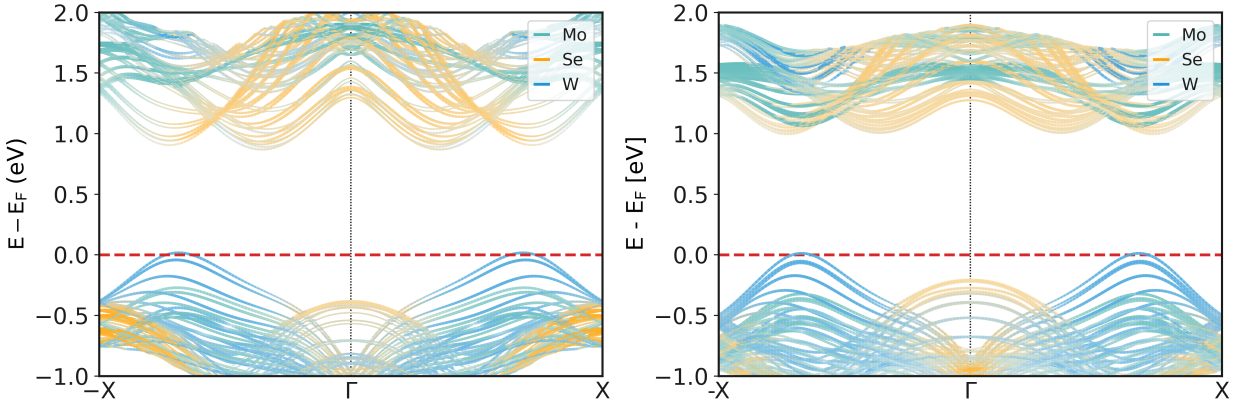

Figure S23: Wrinkling relaxes some of the strain in the heterobilayer $WSe_2/MoSe_2$ as evident from the changed K-Q separation in the conduction band. The band structure of heterobilayer $WSe_2/MoSe_2$ at 5% compression for left) without wrinkling (i.e. uniaxial strained) and right) relaxed also in the out of plane direction and wrinkles are formed

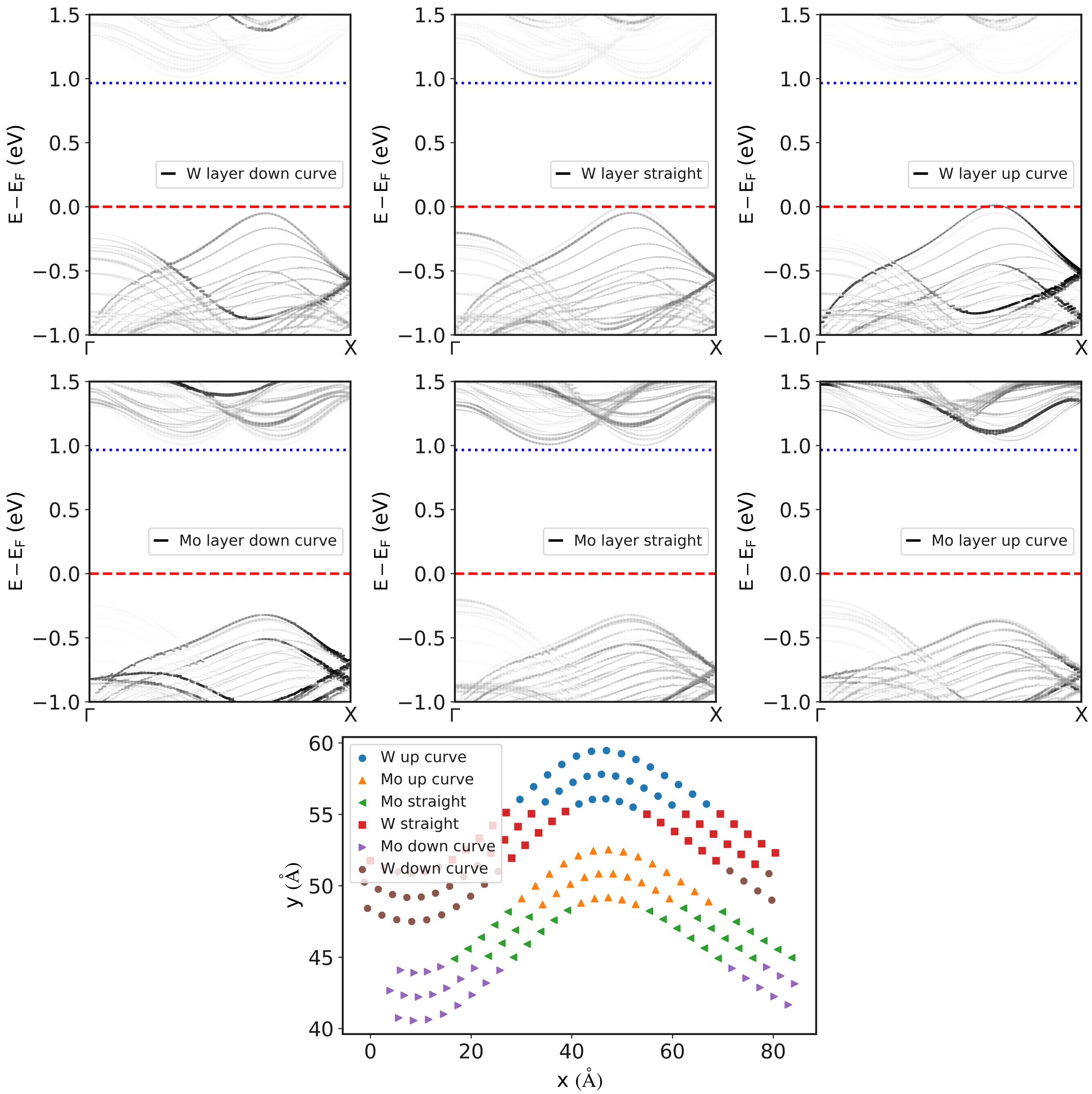

Figure S24: Band structure of $WSe_2/MoSe_2$ heterobilayer at 2.5% compression projected on different sections of the wrinkle, the location of VBM (red) and CBM (blue) are also indicated to help the eye, additionally (below) the position of contributing atoms to the band projection is given.

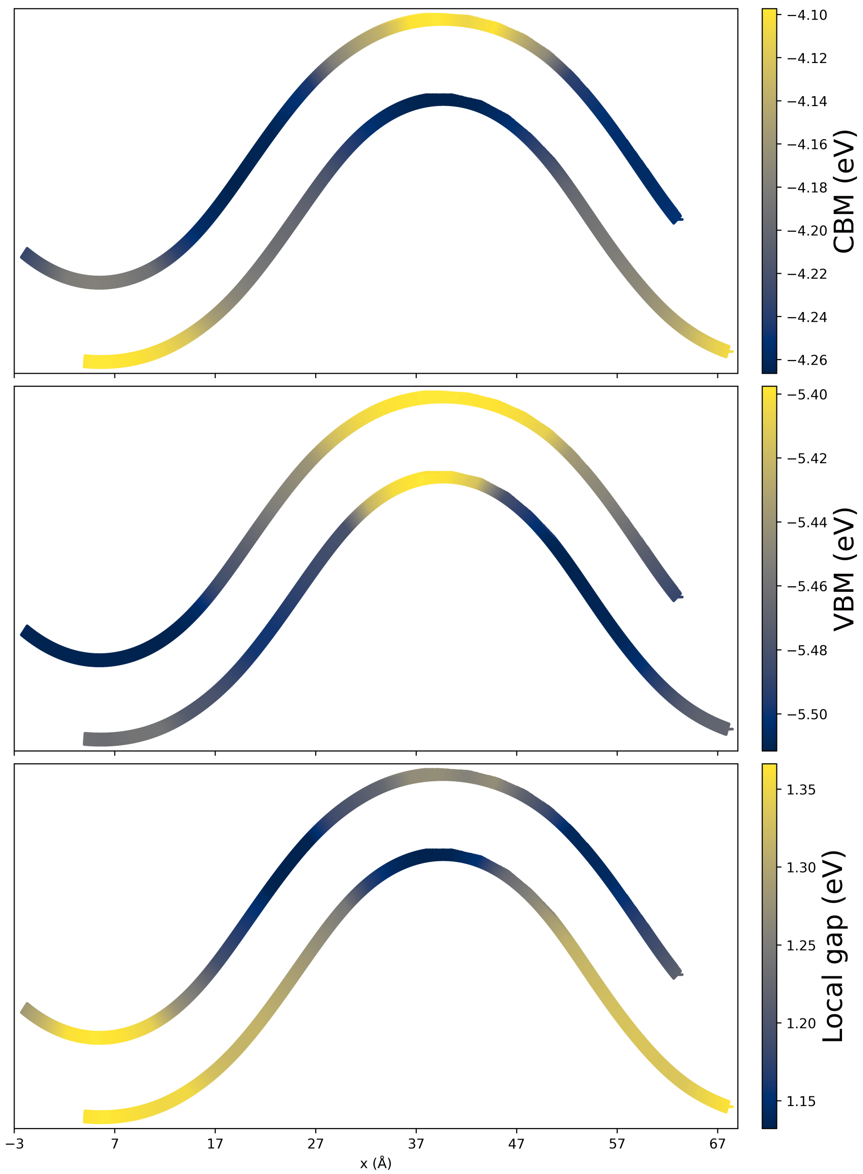

Figure S25: Local distribution of band gap and electronic structure edges (CBM and VBM) of 20% compressed wrinkled heterobilayer of $WSe_2/MoSe_2$. Upper layer: WSe_2 Lower layer: $MoSe_2$. The values are extracted from metal atoms and linearly extrapolated along the wrinkle.

Spin texture in $WSe_2/MoSe_2$ heterobilayer wrinkles

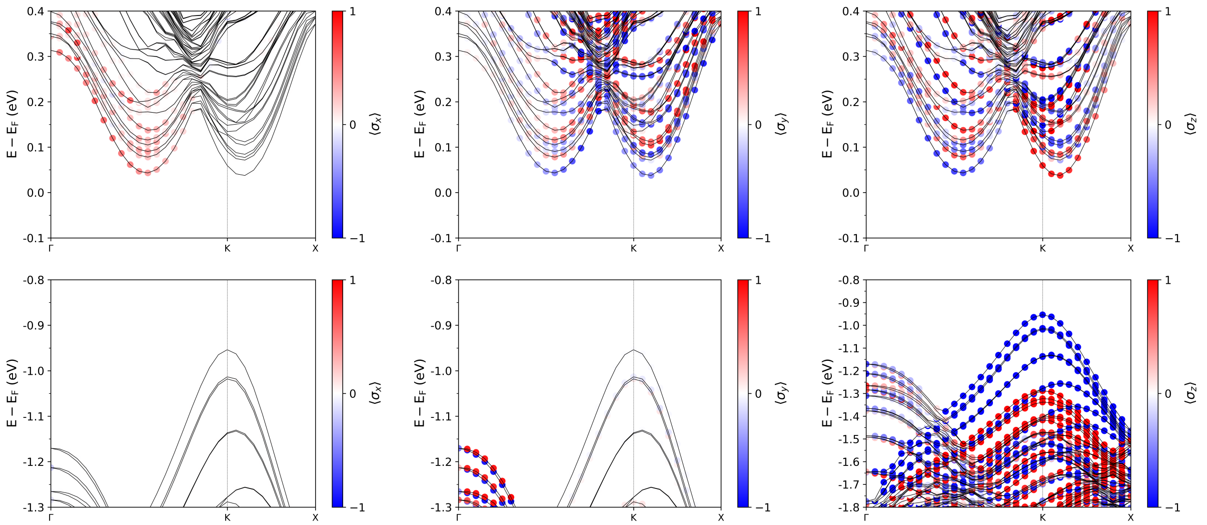

Figure S26: Expectation values of the Pauli matrices $\langle\sigma_i\rangle$ of the wrinkled $WSe_2/MoSe_2$ at 2.5% compression

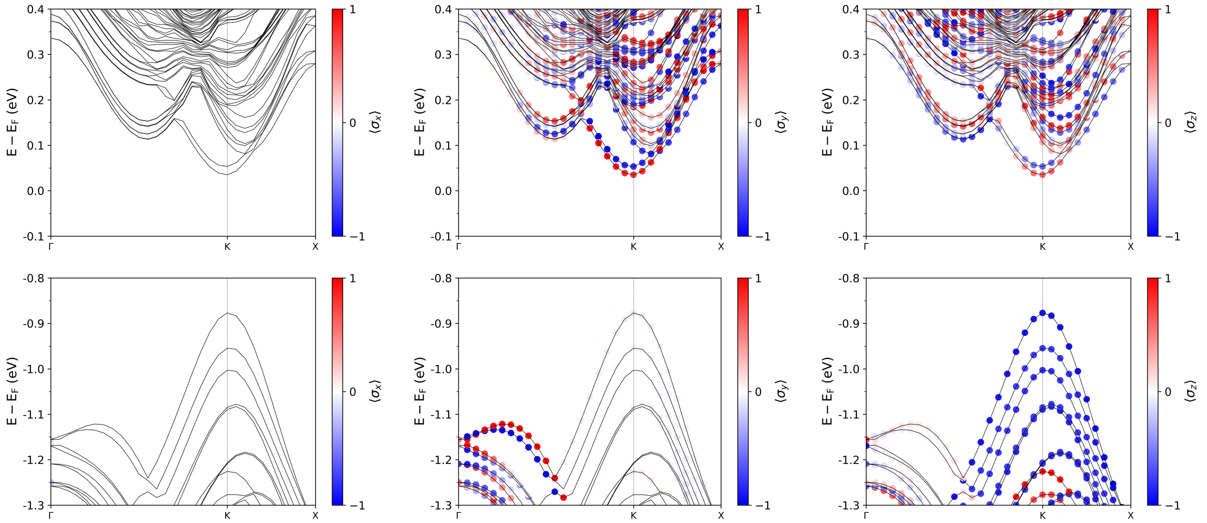

Figure S27: Expectation values of the Pauli matrices $\langle\sigma_i\rangle$ of the wrinkled $WSe_2/MoSe_2$ at 20% compression, red (blue) line indicates VBM (CBM) energies to help the eye

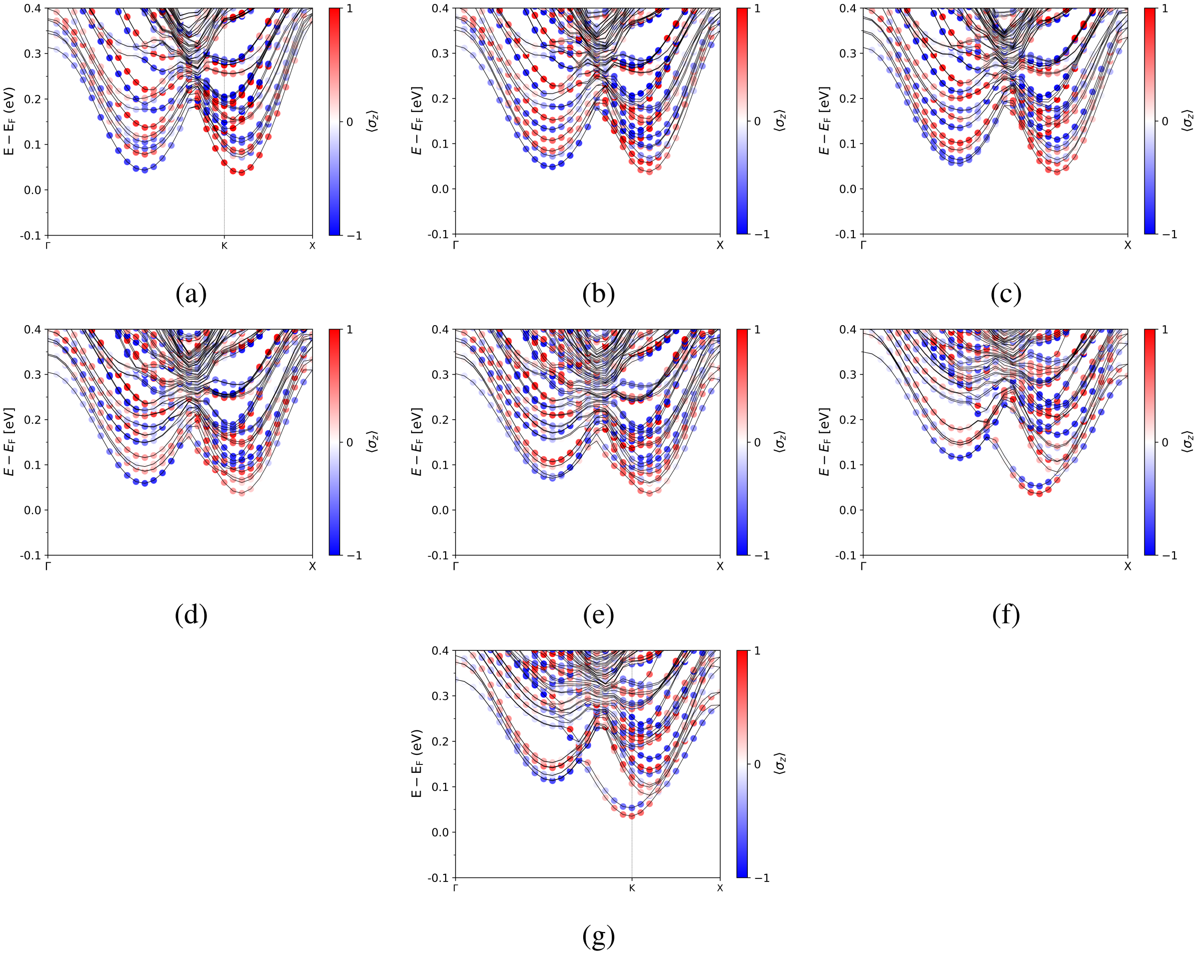

Figure S28: Z component of the expectation values of the Pauli matrices $\langle \sigma_z \rangle$ for the lowest conduction bands (CB) of the wrinkled $WSe_2/MoSe_2$ at different strain a) 2.5 % b) 5% c) 7.5 % d) 10% e) 12.5 % 15 % f) 17.5 % g) 20 %

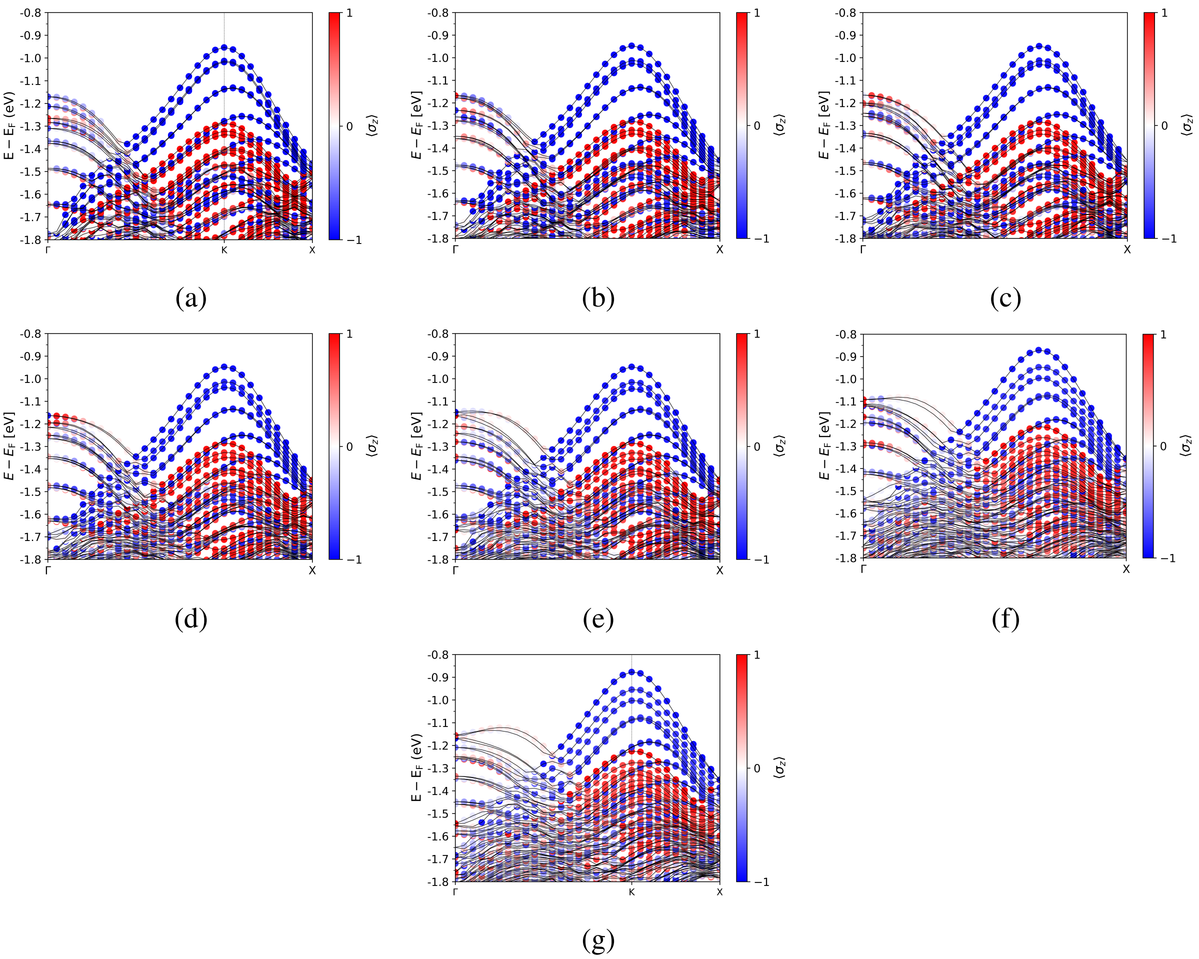

Figure S29: Z component of the expectation values of the Pauli matrices $\langle \sigma_z \rangle$ for the highest valence bands (VB) of the wrinkled $WSe_2/MoSe_2$ at different strain a) 2.5% b) 5% c) 7.5% d) 10% e)12.5 f) 17.5% g) 20%

Table S11: Maximum and minimum strain at each layer of the wrinkled heterobilayer $WSe_2/MoSe_2$ structure, defined as $\varepsilon = \frac{d-d_{flat}}{d_{flat}}$, where d is the M−M distance and M stands for Mo or W

**Compression**	**Maximum strain Mo layer**	**Minimum strain Mo layer**	**Maximum strain W layer**	**Minimum strain W layer**
2.5	0.0005	-0.0064	0.0005	-0.0064
5	0.0027	-0.0049	0.0027	-0.0049
7.5	0.0011	-0.0080	0.0010	-0.0080
10	0.0038	-0.0070	0.0038	-0.0070
12.5	0.0064	-0.0088	0.0064	-0.0087
15	0.0114	-0.0042	0.0114	-0.0042
17.5	0.0143	-0.0041	0.0143	-0.0041
20	0.0135	-0.0070	0.0135	-0.0070

Table S12: Different band gaps in eV of heterobilayer $WSe_2/MoSe_2$ wrinkles at the backfolded K point for different compression (at least 50 % localization).

Compression	Interlayer band gap	The smallest band gap	W intralayer band gap	Mo intralayer band gap
2.5	1.083	1.017	1.362	1.417
5	1.053	1.002	1.330	1.387
7.5	1.068	1.004	1.340	1.403
10	1.031	1.002	1.365	1.369
12.5	1.024	1.003	1.355	1.362
15	0.923	0.923	1.190	1.264
17.5	0.907	0.907	1.171	1.249
20	0.912	0.912	1.183	1.261

Table S13: Structural parameters of the heterobilayer $WSe_2/MoSe_2$ in Å for different compressions- A and R values are extracted using the fitted curve explained in the SI

Compression	λ	$A_{W_{layer}}$	$A_{Mo_{layer}}$	A_{both}	$R_{min_{W_{layer}}}$	$R_{min_{Mo_{layer}}}$
2.5	83.137	4.309	4.308	7.793	32.192	27.348
5	81.005	6.095	6.103	9.555	28.072	33.868
7.5	78.874	7.382	7.394	10.829	22.201	27.537
10	76.741	8.506	8.52	11.945	18.248	16.35
12.5	74.610	9.473	9.487	12.886	15.733	13.707
15	72.479	10.158	10.14	13.541	8.201	11.219
17.5	70.347	10.944	10.923	14.326	11.576	9.765
20	68.215	11.57	11.526	15.047	10.273	8.625

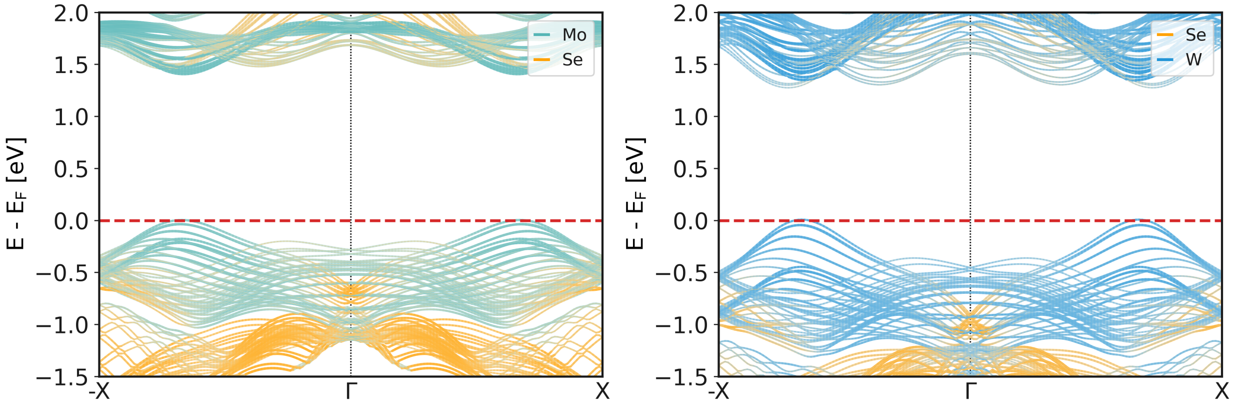

Figure S30: The heterobilayer reduces the Rashba-like splitting in the wrinkle heterobilayer $WSe_2/MoSe_2$. The band structure of the separated layer geometry of the 12.5% compressed heterobilayer $WSe_2/MoSe_2$. Left) $MoSe_2$ and right) WSe_2

Heterobilayer WS_2/MoS_2

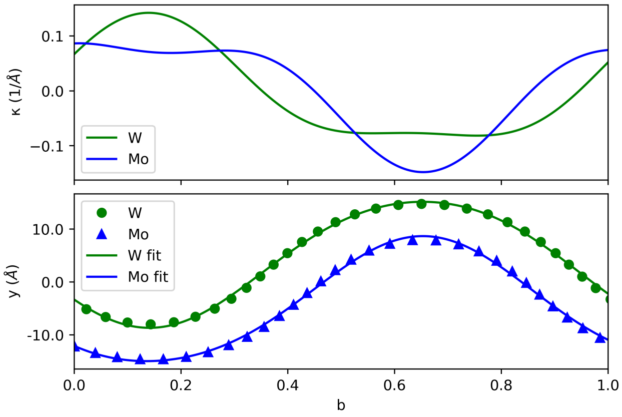

Figure S31: Structural information of wrinkled heterobilayer WS_2/MoS_2. Curvature, κ , and the atomic positions of the W atoms of wrinkled heterobilayer WS_2/MoS_2(lower panel) for 20% compression. b is the unit vector of the lattice parameter in the direction of the wrinkle.

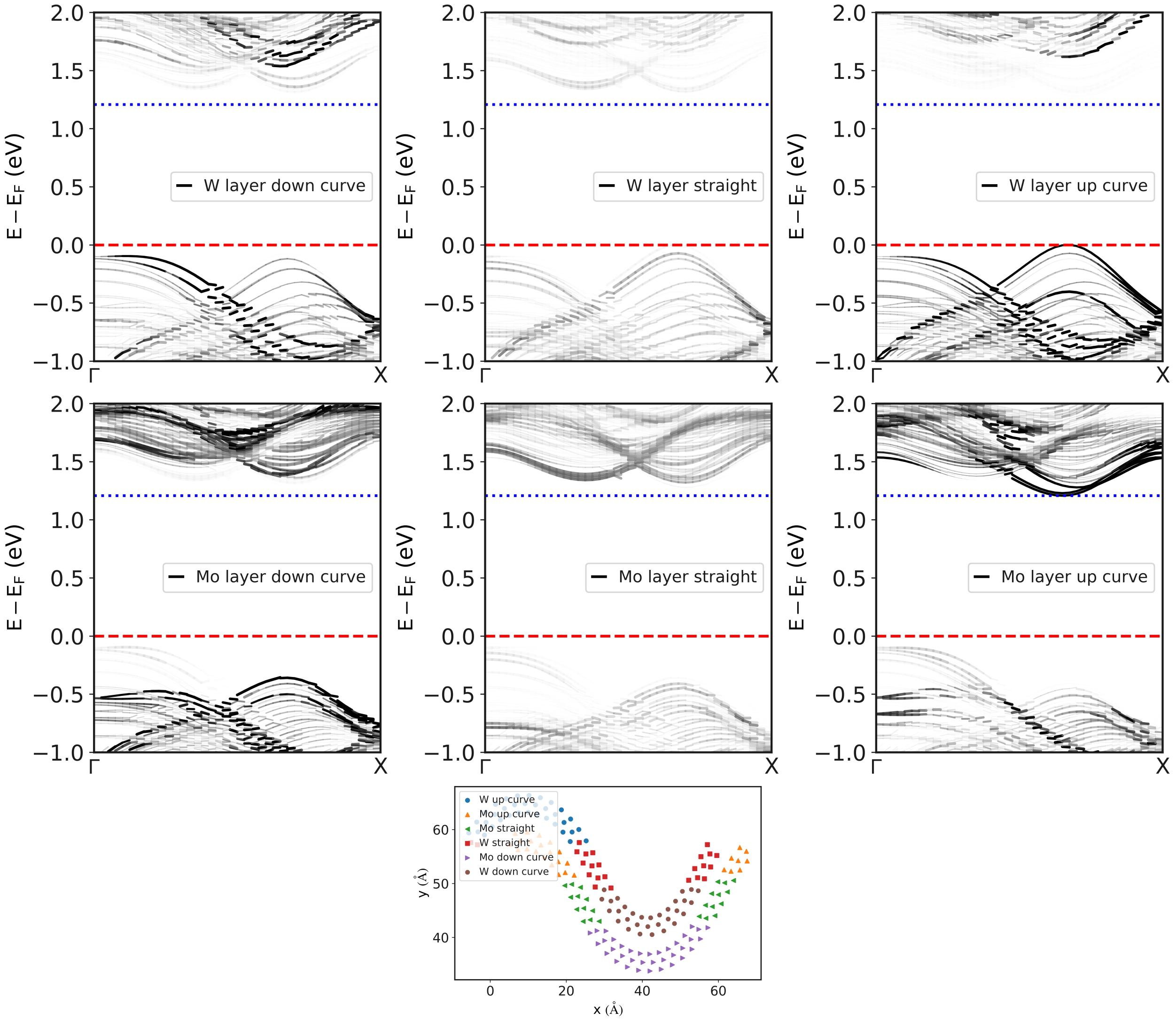

Figure S32: The section projected of the states for the wrinkled heterobilayer WS_2/MoS_2 at 20% compression with the position of contributing atoms.

References

1. Debbichi, L.; Eriksson, O.; Lebègue, S. Electronic structure of two-dimensional transition metal dichalcogenide bilayers from ab initio theory. *Physical Review B* **2014**, *89*, 205311.

2. Komsa, H.-P.; Krasheninnikov, A. V. Electronic structures and optical properties of realistic transition metal dichalcogenide heterostructures from first principles. *Physical Review B—Condensed Matter and Materials Physics* **2013**, *88*, 085318.

3. Desai, S. B.; Seol, G.; Kang, J. S.; Fang, H.; Battaglia, C.; Kapadia, R.; Ager, J. W.; Guo, J.; Javey, A. Strain-induced indirect to direct bandgap transition in multilayer WSe2. *Nano letters* **2014**, *14*, 4592–4597.

4. Manchon, A.; Koo, H. C.; Nitta, J.; Frolov, S. M.; Duine, R. A. New perspectives for Rashba spin–orbit coupling. *Nature materials* **2015**, *14*, 871–882.

5. Daqiqshirazi, M.; Brumme, T. Funneling and spin-orbit coupling in transition metal dichalcogenide nanotubes and wrinkles. *Physical Review B* **2023**, *108*, 155304.

6. Yu, H.; Liu, G.-B.; Yao, W. Brightened spin-triplet interlayer excitons and optical selection rules in van der Waals heterobilayers. *2D Mater.* **2018**, *5*, 035021.

7. Woźniak, T.; Faria Junior, P. E.; Seifert, G.; Chaves, A.; Kunstmann, J. Exciton *g* factors of van der Waals heterostructures from first-principles calculations. *Phys. Rev. B* **2020**, *101*, 235408.

8. Jones, A. M.; Yu, H.; Ross, J. S.; Klement, P.; Ghimire, N. J.; Yan, J.; Mandrus, D. G.; Yao, W.; Xu, X. Spin-layer locking effects in optical orientation of exciton spin in bilayer WSe2. *Nature Physics* **2014**, *10*, 130–134.
